# Supplementary figures and images for: Multiomics in silico analysis identifies TM4SF4 as a cell surface target in hepatocellular carcinoma
Source: PLoS One. 2025 Feb 25;20(2):e0307048. doi: 10.1371/journal.pone.0307048 (PMC11856526; doi:10.1371/journal.pone.0307048)

S1 Fig

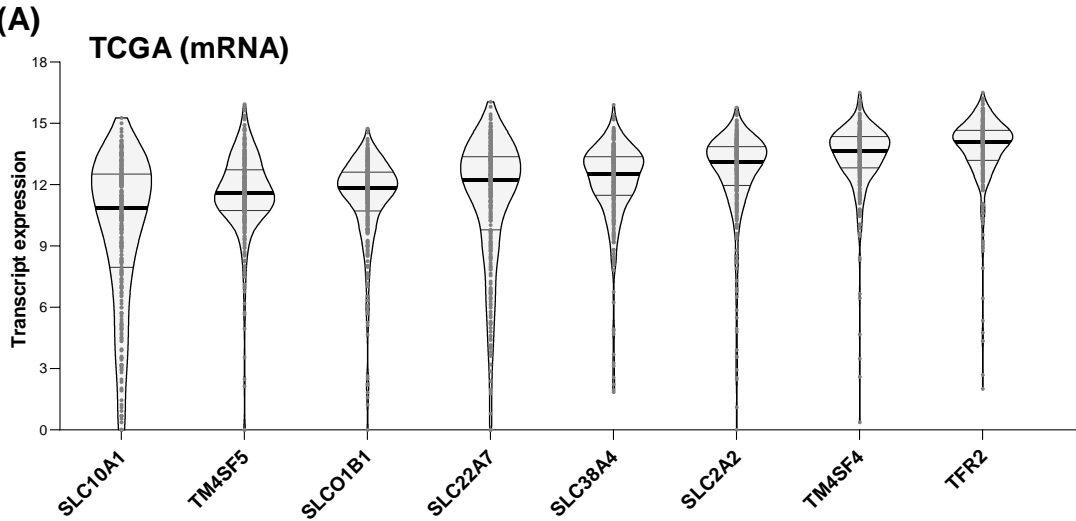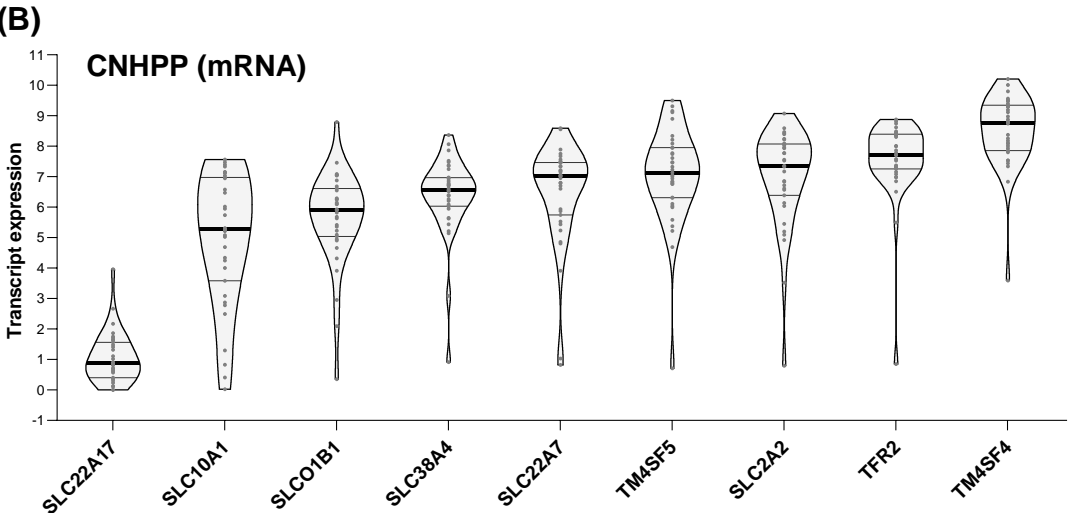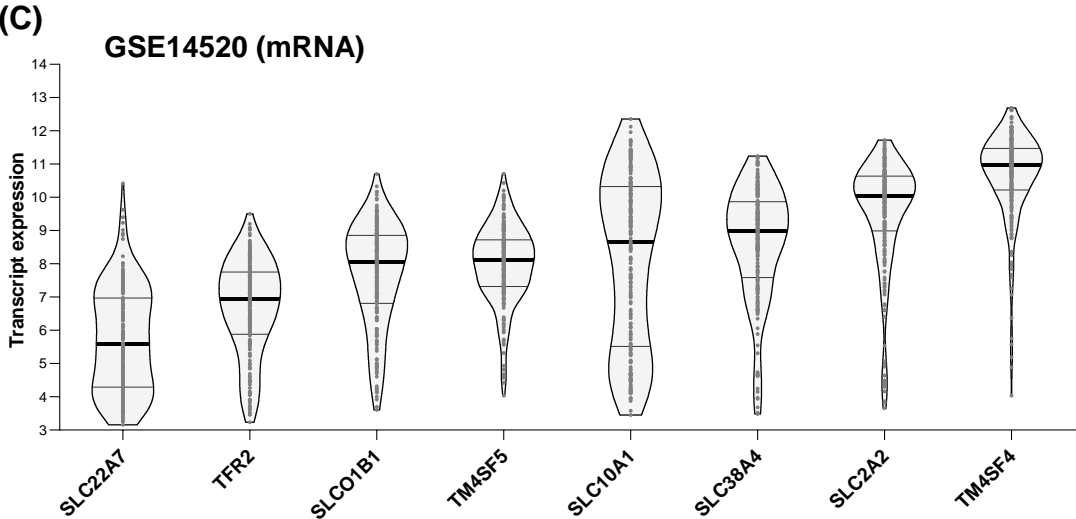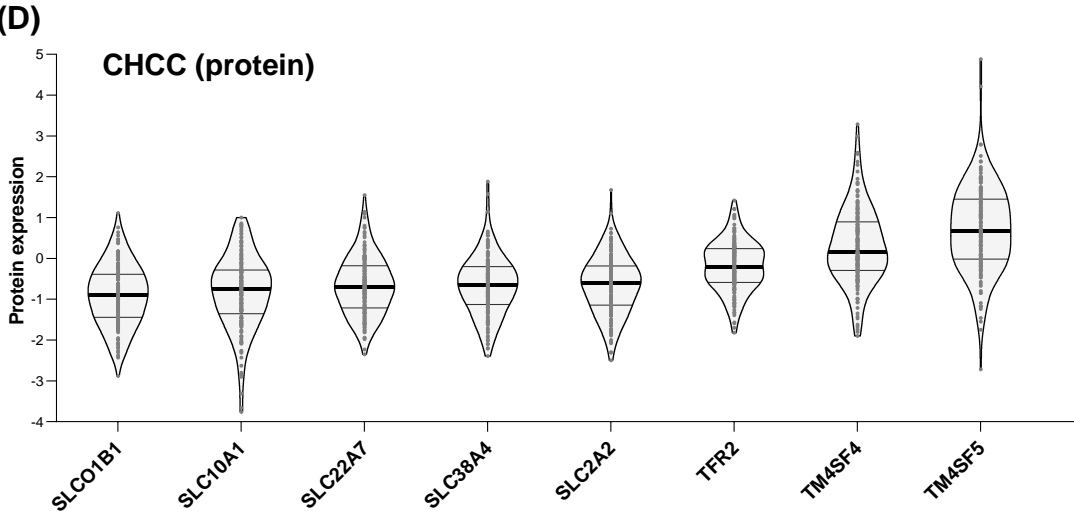

Supplement: S1 Fig — Violin plots of the transcript expression levels in TCGA (n = 366) (A), CNHPP (n = 35) and GSE14520 (n = 225) HCC cases, and the protein expression levels in CHCC (n = 165) HCC cases (D). The violin plots within each graph were arranged from left to right according to ascending median transcript or protein expression values for each gene. For each violin plot, the thick black bar in the center denotes the median while the upper and lower lines denote the third and first quartile, respectively. (PDF) [file pone.0307048.s001.pdf]

S2 Fig

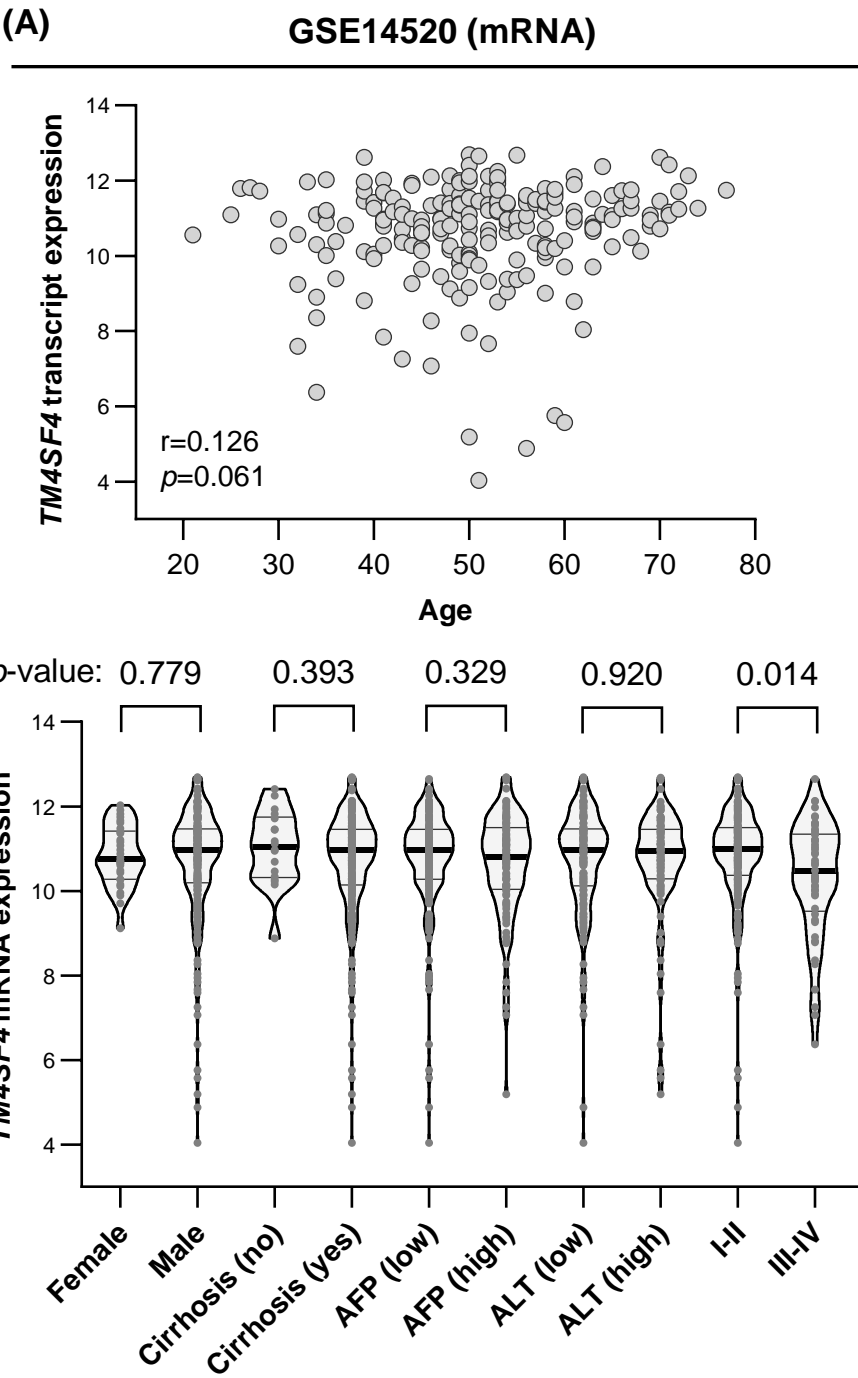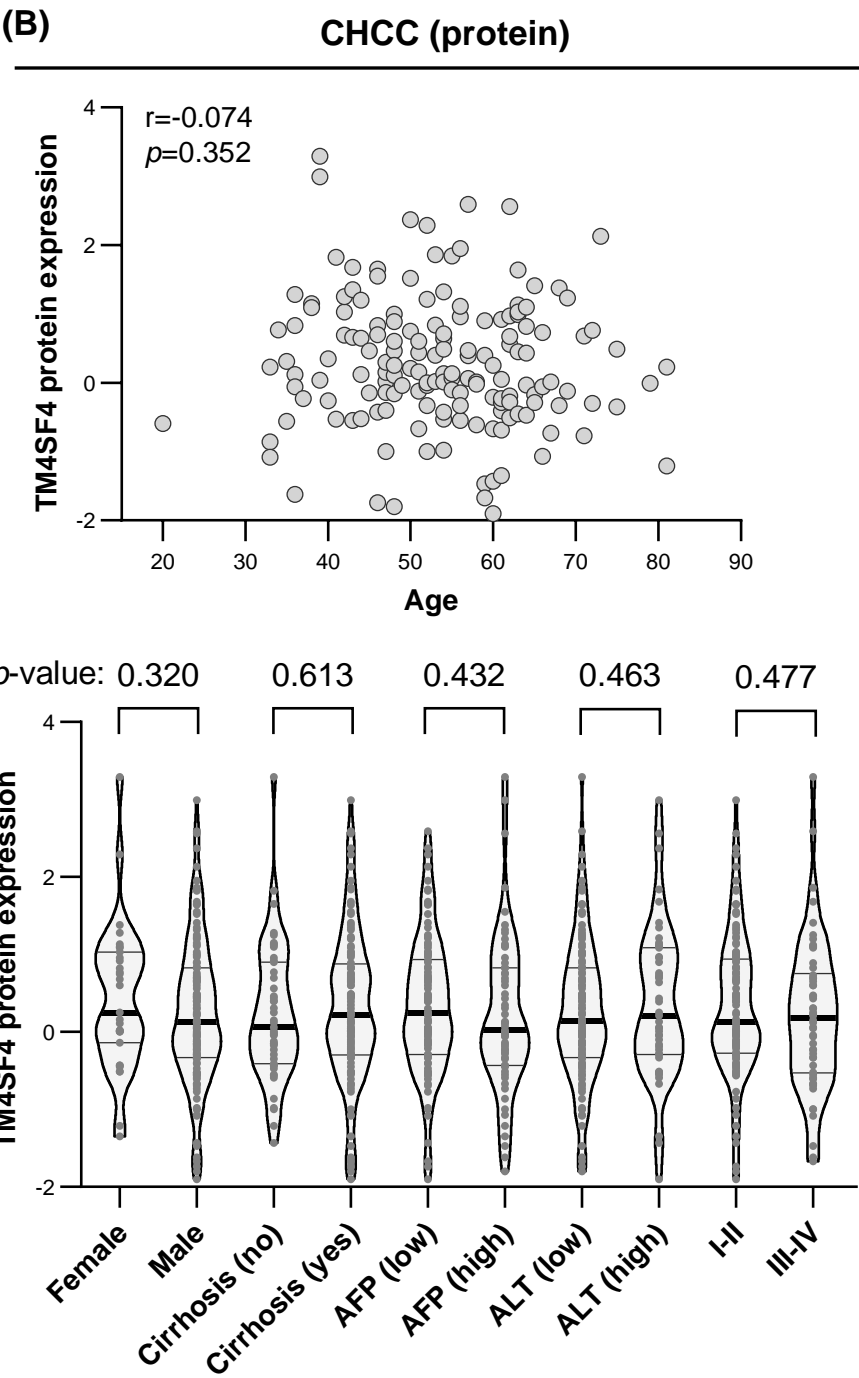

Supplement: S2 Fig — (A) Association of TM4SF4 transcript levels with clinico-demographic characteristics of HCC patients in the GSE14520 dataset (n = 221); (B) Association of TM4SF4 protein levels with clinico-demographic characteristics of HCC patients in the CHCC dataset (n = 159). (PDF) [file pone.0307048.s002.pdf]

S3 Fig

(A)

GSE14520 (mRNA)

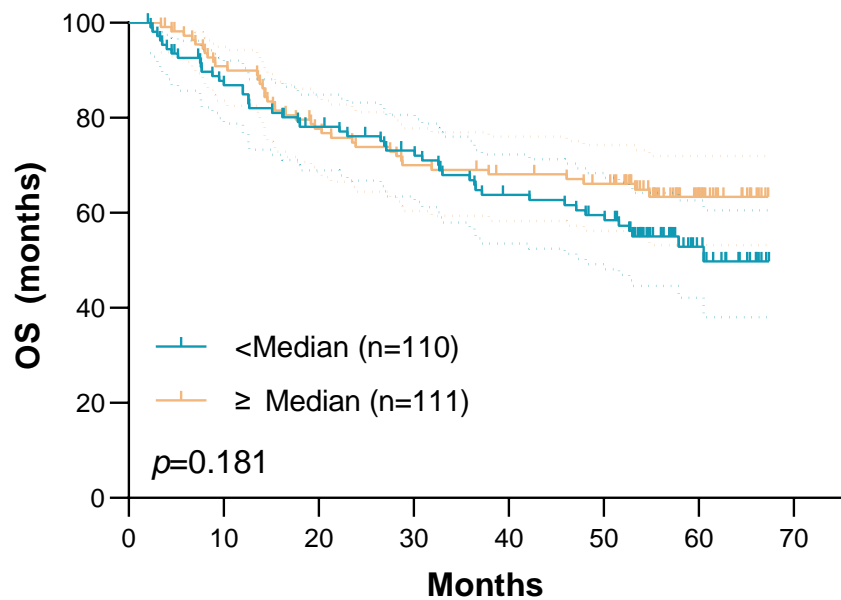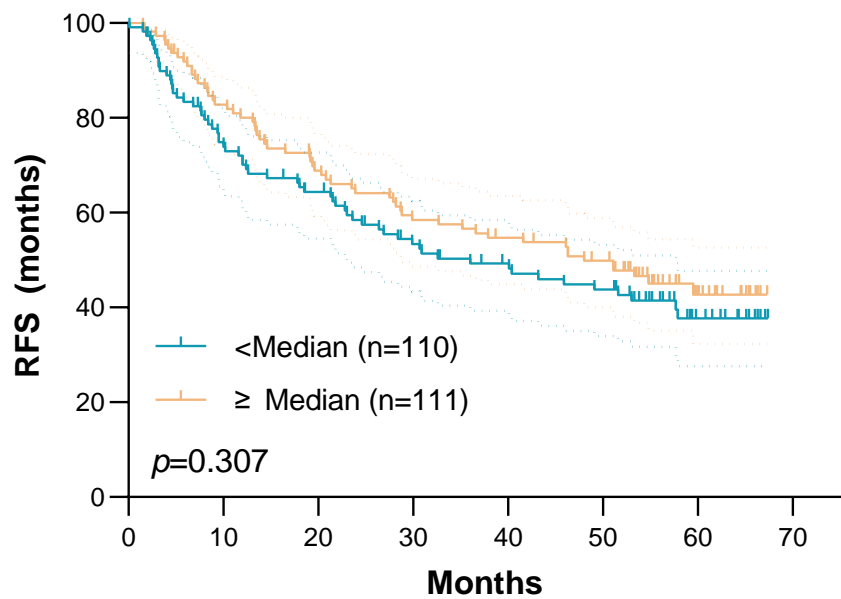

(B)

CHCC (protein)

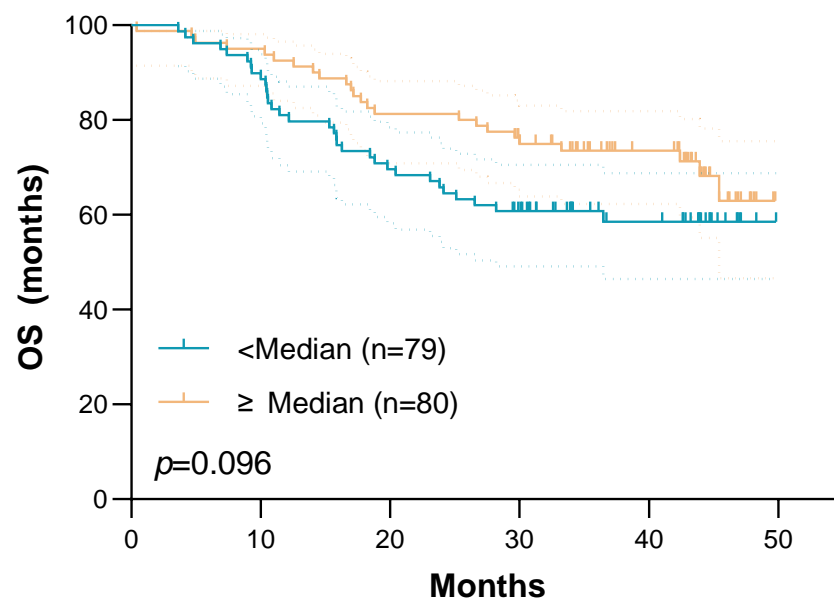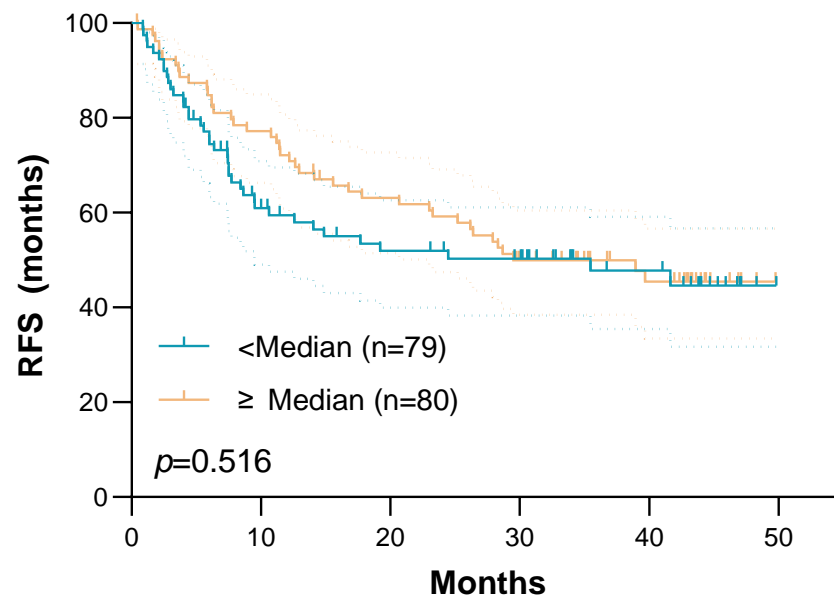

Supplement: S3 Fig — (A) Association of TM4SF4 transcript levels with the survival of HCC patients in the GSE14520 dataset (n = 221); (B) Association of TM4SF4 protein levels with the survival of HCC patients in the CHCC dataset (n = 159). (PDF) [file pone.0307048.s003.pdf]
