# Supplementary material for: Multiomics in silico analysis identifies TM4SF4 as a cell surface target in hepatocellular carcinoma
Source: PLoS One. 2025 Feb 25;20(2):e0307048. doi: 10.1371/journal.pone.0307048 (PMC11856526; doi:10.1371/journal.pone.0307048)
Supplement: S4 Table — (DOCX) [file pone.0307048.s007.docx]

**Table S4** Normal functions and potential association with TM4SF4 of the genes highly correlated with *TM4SF4* expression in HCC cells (n=32).

| **Gene** | **Normal Functions** | **Potential Association with TM4SF4** | **TCGA (GEPIA2)** |
| --- | --- | --- | --- |
| ***CLU*** | - Clusterin (CLU) is an ATP-independent molecular chaperone activated by cellular stress, and it is required for the homeostasis of proteins as well as inhibition of cell death pathways by inducing pro-survival signaling and transcriptional network [1]. | - *CLU* and *TM4SF4* were predicted to be target genes of CDX1, a transcription factor in intestines and colon cancer [2], in Barrett’s esophagus which is a pathologic condition that can progress into malignancy [3]. | HCC: 4,368.43  Normal: 2,947.6  *p*-value: NS |
| ***GC*** | - GC vitamin D binding protein (GC) is a main plasma carrier for circulating vitamin D and its metabolites, and it is expressed in the liver that aids in the conversion of sterol derivatives into pre-hormone 25-OH vitamin D [4]. | - GC is co-expressed with TM4SF4 in pancreatic α cells [5]. GC forms part of the network with TM4SF4 in the STRING database (default settings without any modification; <https://string-db.org/>) due to co-expression [6]. | HCC: 1,569.95  Normal: 1,315.94  *p*-value: NS |
| ***SCP2*** | - Sterol carrier protein 2 (SCP2) is an intracellular cholesterol trafficking protein that targets cholesterol to cholesterol-rich, membrane microstructural domains [7-9]. | - *SCP2* and *TM4SF4* were part of 719 specific genes associated with liver metastasis of colon carcinoma [10]. | HCC: 221.83  Normal: 304.23  *p*-value: NS |
| ***AGT*** | - Angiotensinogen (AGT) serves as the sole precursor of the renin-angiotensin system (RAS), producing all angiotensin peptide products via sequential enzymatic cleavages. As such, AGT reflects the activity and status of the entire RAS [11]. | - *Agt* and *Tm4sf4* were downregulated by tauroursodeoxycholic acid, an anti-hepatocarcinogenesis compound [12], in rat hepatocytes [13]. - *Agt* and *Tm4sf4* were markers of diabetic nephropathy in animal models [14]. | HCC: 1,058.79  Normal: 692.23  *p*-value: NS |
| ***TMBIM6*** | - Transmembrane BAX inhibitor motif containing 6 (TMBIM6) is an anti-apoptotic protein that inhibits Bax activation and prevents its translocation to the mitochondria, protecting cells from apoptosis. It is primarily localized on the surface of the endoplasmic reticulum, where it regulates Ca²⁺ transfer from the endoplasmic reticulum to the cytosol and mitochondria [15, 16]. | - TMBIM6 and TM4SF4 had been identified as interactors with GLP1R, a pancreatic islets hormonal receptor that plays key roles in maintaining glucose homeostasis by regulating the secretion of insulin and glucagon, in human pancreatic islet cells [17]. | HCC: 494.76  Normal: 387.61  *p*-value: NS |
| ***ALB*** | - Albumin (ALB) is produced in the liver and it is the most abundant plasma protein in healthy individuals. It plays key roles in diverse processes including plasma colloid osmotic pressure maintenance, transport of endogenous or exogenous molecules, stabilization of endothelial, and with anti-thrombosis and anti-inflammation properties [18]. | - *ALB* and *TM4SF4* were two of six genes upregulated >50-fold in early-stage non‑small cell lung adenocarcinoma [19]. | HCC: 27,848.27  Normal: 30,636.39  *p*-value: NS |
| ***SDHC*** | - Succinate dehydrogenase complex subunit C (SDHC) forms a subunit of mitochondrial complex II (also known as succinate dehydrogenase, SDH, or succinate), representing the smallest respiratory complex of the respiratory chain. SDHC is anchored to the membrane of SDH, connecting the tricarboxylic acid cycle to the electron transport chain, and involved in cellular energy generation as well as apoptosis [20]. | - *SDHC* and *TM4SF4* were part of 456 genes upregulated in intestinal metaplasia [21]. - Sdhc and Tm4sf4 were both upregulated by the overexpression of liver X receptor alpha (LXRα), a nuclear receptor involved in lipid metabolism and anti-inflammatory processes, in xenograft tumors (HT29 colorectal cancer cells overexpressing LXRα) [22]. | HCC: 236.14  Normal: 134.49  *p*-value: NS |
| ***TMEM176B*** | - Transmembrane protein 176B (TMEM176B) is a membrane-bound protein localized to endosomes, where it mediates immune responses, inflammasome activation, and in the development and regulation of myeloid immune cells. Additionally, TMEM176B maintains immune homeostasis by regulating immune cell infiltration and activation [23]. | - *TMEM176B* and *TM4SF4* were part of 16 genes upregulated in human non-diabetic islet α cells [24]. This was also observed in another study where both genes were predictive of human non-diabetic islet α cells [25]. | HCC: 1,061.95  Normal: 740.8  *p*-value: NS |
| ***ACAA2*** | - Acetyl-coenzyme A acyltransferase 2 (ACAA2) is a mitochondrial thiolase enzyme involved in medium-chain fatty acid metabolism, and it also acts as a thyroid hormone-dependent coactivator of thyroid hormone receptor beta-1 (TRβ1) in the heart [26]. | - *ACAA2* was downregulated in HBV-positive HCC cases, while *TM4SF4* was upregulated in HCV-positive HCC cases [27]. - *ACAA2* and *TM4SF4* were part of 456 genes upregulated in intestinal metaplasia [21] in the same study as described for *SDHC* and *TM4SF4* above. | HCC: 158.88  Normal: 292.73  *p*-value: NS |
| ***RARRES2*** | - Retinoic acid receptor responder 2 (RARRES2) encodes chemerin, a chemoattractant and adipokine highly expressed in adipose tissue and it functions to mediate adipogenesis and adipocyte homeostasis [28, 29]. | - *RARRES2* and *TM4SF4* were differentially expressed in HuH-7 cell line (HCC cells) transfected with recombinant HCV-1b core shadow protein [30]. | HCC: 1,278.91  Normal: 874.83  *p*-value: NS |
| ***AMBP*** | - Alpha-1-microglobulin/bikunin precursor (AMBP) is a hepatic secretory protein filtered by the glomerulus and reabsorbed by proximal tubular epithelial cells (PTECs), where it displays radical-scavenging and heme-binding activities that protect PTECs from oxidative damage [31]. | - *AMBP* and *TM4SF4* were part of 33 feature genes that predicted liver metastasis of colorectal adenocarcinoma, and both were also involved in protein-protein interaction network suggesting interactions between the proteins [32]. - *Ambp* and *Tm4sf4* were both expressed in a specific cluster of hepatocytes (Hepatoycyte-3 cluster) by scRNA-seq of mice hepatic macrophages [33]. | HCC: 3,932.41  Normal: 2,516.96  *p*-value: NS |
| ***MPC2*** | - Mitochondrial pyruvate carrier 2 (MPC2) is a subinit of the mitochondrial pyruvate carrier complex, and it is required to import pyruvate into the mitochondrial matrix, leading to glycolysis association with oxidative phosphorylation [34]. | - *MPC2* and *TM4SF4* were both downregulated in human epithelial cells (including H1299, Caco2, and Calu3 cell lines) infected by SARS-CoV-2 versus controls [35, 36]. | HCC: 279.05  Normal: 110.93  ***p*-value: <0.001** |
| ***NDUFC2*** | - NDUFC2 is a key subunit for the function of complex I in the mitochondrial respiratory chain, required to maintain mitochondrial functions and to protect against oxidative stress. As such NDUFC2 has protective effects against cardiovascular events, highlighting its roles in mitochondrial and cellular homeostasis [37]. | - No potential link with TM4SF4 was found | HCC: 323.85  Normal: 140.87  ***p*-value: <0.001** |
| ***NDUFB2*** | - NDUFB2 is another subunit of mitochondrial CI, where it facilitates electron transfer from NADH to the respiratory chain, playing key roles in oxidative phosphorylation and cellular metabolism. NDUFB2 is also involved in DNA repair, cell cycle regulation, and inflammation regulation [38]. | - No potential link with TM4SF4 was found | HCC: 230.58  Normal: 129.38  *p*-value: NS |
| ***ECH1*** | - Enoyl coenzyme A hydratase 1 (ECH1) is a mitochondrial enzyme involved in fatty acid β-oxidation and lipid metabolism. ECH1 also protects against nonalcoholic steatohepatitis by inhibiting ferroptosis, a form of iron-dependent cell death associated with lipid peroxidation and oxidative stress [39]. | - *ECH1* and *TM4SF4* were part of 719 specific genes associated with liver metastasis of colon carcinoma [10] in the same study as described above for *SCP2* and *TM4SF4*. | HCC: 349.03  Normal: 259.26  *p*-value: NS |
| ***VTN*** | - Vitronectin (VTN) is a multifunctional adhesive glycoprotein found in serum, extracellular matrix, and bone. VTN plays key roles in cell adhesion, differentiation, and angiogenesis by activating downstream signaling pathways such as VEGFR2 [40, 41]. | - *Vtn* and *Tm4sf4* were each upregulated in bone and liver metastasis, respectively, of non-small cell lung cancer in mice [42]. | HCC: 2,930.84  Normal: 1,860.21  *p*-value: NS |
| ***GSTK1*** | - Glutathione S-transferase kappa 1 (GSTK1) is a mitochondrial enzyme in the GST family that detoxifies xenobiotics, environmental carcinogens, and reactive oxygen species by catalyzing the conjugation of glutathione to hydrophobic compounds. GSTK1 protects against oxidative stress, preserving cellular macromolecules and supporting the cellular adaptive response [43]. | - *Gstk1* and *Tm4sf4* were both involved in response to liver injury and metabolic stress in mice, where *Gstk1* downregulation and *Tm4sf4* upregulation were mitigated by probiotics in acetaminophen-induced liver injury [44]. - *Gstk1* and *Tm4sf4* were each downregulated and upregulated, respectively, in HCC (expressing SRF-VP16 transgene, an oncoprotein involved in Ras/MAPK and Rho/actin signaling in HCC) vs controls (liver tissues from mice without SRF-VP16 transgene) [45] | HCC: 279.98  Normal: 194.9  *p*-value: NS |
| ***MGST3*** | - Microsomal glutathione transferase 3 (MGST3) regulates the metabolism of eicosanoid and glutathione, associated with oxidative stress and cellular apoptosis [46]. | - *Mgst3* and *Tm4sf4* were both upregulated by 3,5-diethoxycarbonyl-1,4-dihydrocollidine (DDC), a compound used to induce cholestatic disease in mice, in DDC-induced cholestatic liver disease [47]. - *GST3* and *TM4SF4* expressions were altered by perfluorinated chemicals, organic pollutants that induce hepatotoxic effects, in human hepatoma cell line HepG2 [48]. | HCC: 123.34  Normal: 67.7  *p*-value: NS |
| ***COX6A1*** | - Cytochrome c oxidase subunit 6A1 (COX6A1) is an anti-apoptosis factor involved in the autophagy-lysosome pathway [49]. | - *COX6A1* and *TM4SF4* predicted to be targeted by hsa-miR-99a and hsa-miR-192, respectively, and these miRNAs are expressed in human fetal liver [50]. - Both genes shown to be differentially expressed in acute myeloid leukemia with inv(16)(p13q22) (also known as M4Eo) [51]. | HCC: 639.1  Normal: 304.8  ***p*-value: <0.001** |
| ***PRDX6*** | - Peroxiredoxin 6 (PRDX6) belongs to a family of enzymes, the peroxiredoxins (PRDXs), that primarily act as antioxidants to scavenge peroxide. - PRDX6 has been shown to have multiple functions including redox homeostasis, lipids turnover or metabolism, and cellular signaling [52]. | - *Prdx6* and *Tm4sf4* expressions were regulated by Ehmt2, directly or indirectly, in young adult mice associated with immune responses in acute pancreatitis [53]. | HCC: 403.55  Normal: 254.48  *p*-value: NS |
| ***TMCO1*** | - Transmembrane coiled-coil domains 1 (TMCO1) regulates the levels of endoplasmic reticulum Ca^2+^ by facilitating Ca^2+^ loss when there is excessive endoplasmic reticulum Ca^2+^ store levels [54]. | - *TMCO1* and *TM4SF4* expression was downregulated and upregulated, respectively, by the treatment of human umbilical vein endothelial cells (HUVECs) with lovastatin, a statin that decreases triglycerides and facilitates cholesterol removal in the liver [55]. | HCC: 124.94  Normal: 41.11  ***p*-value: <0.001** |
| ***COPZ1*** | - Coatomer protein complex zeta 1 (COPZ1) forms the heptameric coatomer protein complex I (COPI), involved in Golgi-related processes such as the assembly of coated vesicles on Golgi membranes and proteins transport in the endoplasmic reticulum-Golgi secretory pathway [56]. | - *Copz1* and *Tm4sf4* were both upregulated in liver of mice exposed to hydrazine (metabolites of the anti-tuberculosis drug isoniazid (INH) and involved in INH-induced liver damage), suggesting that both may be involved in INH-induced liver damage [57]. | HCC: 81.88  Normal: 40.16  ***p*-value: <0.001** |
| ***TMEM176A*** | - Transmembrane domain protein 176A (TMEM176A) is preferentially expressed in myeloid cells and functions to maintain the immature state of dendritic cells [58]. | - Both *TMEM176A* and *TM4SF4* were upregulated in ALDH1-negative/Bmi1-overexpressed head and neck squamous cell carcinoma (HNSCC) compared to ALDH1-negative HNSCC [59]. | HCC: 728.56  Normal: 562.75  *p*-value: NS |
| ***COX8A*** | - Cytochrome c oxidase 8A (COX8A) is the smallest subunit of the COX complex and it is critical for the function of human complex IV where its mutation causes Leigh-like syndrome and epilepsy [60]. | - *Cox8a* and *Tm4sf4* were differentially expressed during hepatic ischemia-reperfusion in rats, with *Cox8a* being differentially expressed in the reperfused lobe at 0 hour of reperfusion, while *Tm4sf4* expression increased in the ischemic lobe at 2 hours of reperfusion [61]. | HCC: 396.56  Normal: 187.07  ***p*-value: <0.001** |
| ***CFH*** | - Complement factor H (CFH) mediates the alternative complement pathway by recognizing self-surfaces via glycosaminoglycans, sialic acid, or C-reactive protein. It also clears cell debris without inducing inflammation and interacts with receptors and proteins like CR3 and apoE [62]. | - *CFH* and *TM4SF4* were among the genes identified in initial screening analyses of pancreatic cancer datasets, but without any other specific or further information of both genes in these cases [63]. - *CFH* and *TM4SF4* were differentially enriched in plasma-derived extracellular vesicles (healthy individuals) using different isolation methods. CFH being enriched in PS-isolated vesicles compared to UC-based methods, while TM4SF4 was enriched in UC3/UC2-qEV-isolated vesicles compared to the basic UC2 method [64]. | HCC: 392.16  Normal: 334.53  *p*-value: NS |
| ***PHYH*** | - Phytanoyl-CoA 2-Hydroxylase (PHYH) is an enzyme within peroxisomes that functions to break down specific branched-chain fatty acids via alpha-oxidation, and it is required for the metabolism of phytanic acid, a dietary fat found in dairy products and some meats [65, 66] | - *PHYH* and *TM4SF4* were identified as components of distinct gene pairs in a 64-gene signature for classifying BRAF-mutant colon cancers, with *PHYH* paired against *DUSP4* and *TM4SF4* paired against *PTPRO* in the diagnostic classifier [67]. | HCC: 149.71  Normal: 113.96  *p*-value: NS |
| ***SEC61B*** | - SEC61 translocon subunit beta (SEC61B) is a subunit of the SEC61 translocon complex required for endoplasmic reticulum protein transport and to stabilize protein translocation [68, 69] | - *SEC61B* and *TM4SF4* were identified as two of 155 interacting partners of Hepatitis E virus non-structural proteins (ORF1 domains) from a yeast two hybrid system screening [70]. - *SEC61B* and *TM4SF4* were identified as two of 31 interactors with GLP1R, receptor for GLP1 that induces insulin secretion, in human pancreatic islets [17]. | HCC: 268.21  Normal: 180.54  *p*-value: NS |
| ***SYPL1*** | - Synaptophysin-like protein 1 (SYPL1) is an integral membrane protein that regulates trans-Golgi network vesicle production required for saccule formation and segregation within the cytoplasmic droplet (CD), controls glycolytic enzyme enrichment in the CD, and maintains protein storage for sperm maturation and fertility [71]. | - *SYPL1* and *TM4SF4* were part of 771 genes upregulated in alcoholic hepatitis, a severe form of alcoholic liver disease with high mortality, compared to control group [72]. | HCC: 39.52  Normal: 20.01  *p*-value: NS |
| ***CARHSP1*** | - Calcium-regulated heat-stable protein 1 (CARHSP1) is a cold-shock domain (CSD) protein family member that acts as a transcriptional or translational regulator, and mediates stability of single-stranded RNA or DNA via its CSD [73, 74] | - *CARHSP1* and *TM4SF4* were both downregulated in epithelial cells derived from SARS-CoV-2 infected samples compared to mock controls (total of 467 downregulated genes) [75]. | HCC: 103.17  Normal: 76.85  *p*-value: NS |
| ***SMIM14*** | - SMIM14 is a type I transmembrane protein found in endoplasmic reticulum, and it is involved in the retention of ER [76]. - It plays multiple roles in the functions of ER such as Ca^2+^ homeostasis and ER stress, as well as regulation of pro-inflammation cytokines secretion and cellular viability [77]. | - *SMIM14* and *TM4SF4* were upregulated or downregulated, respectively, in HCC and alcoholic steatohepatitis cases as shown by analysis of GSE28619 and GSE155907 datasets [78]. | HCC: 36.39  Normal: 47.24  *p*-value: NS |
| ***ATP5A1*** | - ATP synthase F1 subunit alpha (ATP5A1) is a subunit of the mitochondrial ATP synthase that is required to catalyze ATP synthesis and implicated in oncogenesis [79]. | - *Atp5a1 and Tm4sf4* were both upregulated in pigment epithelium-derived factor (PEDF) knockout mice liver, and PEDF is a secreted glycoprotein with antitumor and antiangiogenic properties [80]. | HCC: 237.91  Normal: 163.69  *p*-value: NS |
| ***EDF1*** | - Endothelial differentiation-related factor 1 (EDF1) is required to facilitate ribosome-mediated quality control pathways and to mitigate defective mRNA translation by recruiting translational repressors. The protein also mediates responses to cellular stress by linking cytoplasmic ribosome collision events to transcriptional responses in the nucleus [81]. | - *Edf1* and *Tm4sf4* were upregulated among a longlist of genes in enterocyte differentiation conditions (ENRI media) compared to stem cell conditions (WENRC media) in mouse intestinal mini-gut organoids, suggesting their involvement in enterocyte maturation and differentiation pathways [82]. | HCC: 529.47  Normal: 281.5  *p*-value: NS |

**References**

1. Wilson MR, Zoubeidi A. Clusterin as a therapeutic target. Expert Opin Ther Targets. 2017;21(2):201-13. doi: 10.1080/14728222.2017.1267142. PMID: 27978767.

2. Luk IY, Jenkins LJ, Schoffer KL, Ng I, Tse JWT, Mouradov D, et al. Epithelial de-differentiation triggered by co-ordinate epigenetic inactivation of the EHF and CDX1 transcription factors drives colorectal cancer progression. Cell Death Differ. 2022;29(11):2288-302. doi: 10.1038/s41418-022-01016-w. PMID: 35606410.

3. Wang J, Qin R, Ma Y, Wu H, Peters H, Tyska M, et al. Differential gene expression in normal esophagus and Barrett's esophagus. J Gastroenterol. 2009;44(9):897-911. doi: 10.1007/s00535-009-0082-2. PMID: 19468668.

4. Viloria K, Nasteska D, Briant LJB, Heising S, Larner DP, Fine NHF, et al. Vitamin-D-Binding Protein Contributes to the Maintenance of alpha Cell Function and Glucagon Secretion. Cell Rep. 2020;31(11):107761. doi: 10.1016/j.celrep.2020.107761. PMID: 32553153.

5. Saikia M, Holter MM, Donahue LR, Lee IS, Zheng QC, Wise JL, et al. GLP-1 receptor signaling increases PCSK1 and beta cell features in human alpha cells. JCI Insight. 2021;6(3). doi: 10.1172/jci.insight.141851. PMID: 33554958.

6. Szklarczyk D, Kirsch R, Koutrouli M, Nastou K, Mehryary F, Hachilif R, et al. The STRING database in 2023: protein-protein association networks and functional enrichment analyses for any sequenced genome of interest. Nucleic Acids Res. 2023;51(D1):D638-D46. doi: 10.1093/nar/gkac1000. PMID: 36370105.

7. Burgardt NI, Gianotti AR, Ferreyra RG, Ermacora MR. A structural appraisal of sterol carrier protein 2. Biochim Biophys Acta Proteins Proteom. 2017;1865(5):565-77. doi: 10.1016/j.bbapap.2017.03.002. PMID: 28284963.

8. Schroeder F, Atshaves BP, McIntosh AL, Gallegos AM, Storey SM, Parr RD, et al. Sterol carrier protein-2: new roles in regulating lipid rafts and signaling. Biochim Biophys Acta. 2007;1771(6):700-18. doi: 10.1016/j.bbalip.2007.04.005. PMID: 17543577.

9. Ding X, Fan K, Hu J, Zang Z, Zhang S, Zhang Y, et al. SCP2-mediated cholesterol membrane trafficking promotes the growth of pituitary adenomas via Hedgehog signaling activation. J Exp Clin Cancer Res. 2019;38(1):404. doi: 10.1186/s13046-019-1411-9. PMID: 31519191.

10. Liu J, Wang D, Zhang C, Zhang Z, Chen X, Lian J, et al. Identification of liver metastasis-associated genes in human colon carcinoma by mRNA profiling. Chin J Cancer Res. 2018;30(6):633-46. doi: 10.21147/j.issn.1000-9604.2018.06.08. PMID: 30700932.

11. Xu Y, Rong J, Zhang Z. The emerging role of angiotensinogen in cardiovascular diseases. J Cell Physiol. 2021;236(1):68-78. doi: 10.1002/jcp.29889. PMID: 32572956.

12. Vandewynckel YP, Laukens D, Devisscher L, Paridaens A, Bogaerts E, Verhelst X, et al. Tauroursodeoxycholic acid dampens oncogenic apoptosis induced by endoplasmic reticulum stress during hepatocarcinogen exposure. Oncotarget. 2015;6(29):28011-25. doi: 10.18632/oncotarget.4377. PMID: 26293671.

13. Castro RE, Sola S, Ma X, Ramalho RM, Kren BT, Steer CJ, et al. A distinct microarray gene expression profile in primary rat hepatocytes incubated with ursodeoxycholic acid. J Hepatol. 2005;42(6):897-906. doi: 10.1016/j.jhep.2005.01.026. PMID: 15885361.

14. Hu Y, Kaisaki PJ, Argoud K, Wilder SP, Wallace KJ, Woon PY, et al. Functional annotations of diabetes nephropathy susceptibility loci through analysis of genome-wide renal gene expression in rat models of diabetes mellitus. BMC Med Genomics. 2009;2:41. doi: 10.1186/1755-8794-2-41. PMID: 19586551.

15. Kim HR, Lee GH, Ha KC, Ahn T, Moon JY, Lee BJ, et al. Bax Inhibitor-1 Is a pH-dependent regulator of Ca2+ channel activity in the endoplasmic reticulum. J Biol Chem. 2008;283(23):15946-55. doi: 10.1074/jbc.M800075200. PMID: 18378668.

16. Zhou H, Dai Z, Li J, Wang J, Zhu H, Chang X, et al. TMBIM6 prevents VDAC1 multimerization and improves mitochondrial quality control to reduce sepsis-related myocardial injury. Metabolism. 2023;140:155383. doi: 10.1016/j.metabol.2022.155383. PMID: 36603706.

17. Dai FF, Bhattacharjee A, Liu Y, Batchuluun B, Zhang M, Wang XS, et al. A Novel GLP1 Receptor Interacting Protein ATP6ap2 Regulates Insulin Secretion in Pancreatic Beta Cells. J Biol Chem. 2015;290(41):25045-61. doi: 10.1074/jbc.M115.648592. PMID: 26272612.

18. Sun L, Yin H, Liu M, Xu G, Zhou X, Ge P, et al. Impaired albumin function: a novel potential indicator for liver function damage? Ann Med. 2019;51(7-8):333-44. doi: 10.1080/07853890.2019.1693056. PMID: 31714153.

19. Venugopal N, Yeh J, Kodeboyina SK, Lee TJ, Sharma S, Patel N, et al. Differences in the early stage gene expression profiles of lung adenocarcinoma and lung squamous cell carcinoma. Oncol Lett. 2019;18(6):6572-82. doi: 10.3892/ol.2019.11013. PMID: 31788115.

20. Wang Q, Li M, Zeng N, Zhou Y, Yan J. Succinate dehydrogenase complex subunit C: Role in cellular physiology and disease. Exp Biol Med (Maywood). 2023;248(3):263-70. doi: 10.1177/15353702221147567. PMID: 36691338.

21. Eskandarion MR, Eskandarieh S, Shakoori Farahani A, Mahmoodzadeh H, Shahi F, Oghabian MA, et al. Prediction of novel biomarkers for gastric intestinal metaplasia and gastric adenocarcinoma using bioinformatics analysis. Heliyon. 2024;10(9):e30253. doi: 10.1016/j.heliyon.2024.e30253. PMID: 38737262.

22. Lo Sasso G, Bovenga F, Murzilli S, Salvatore L, Di Tullio G, Martelli N, et al. Liver X receptors inhibit proliferation of human colorectal cancer cells and growth of intestinal tumors in mice. Gastroenterology. 2013;144(7):1497-507, 507 e1-13. doi: 10.1053/j.gastro.2013.02.005. PMID: 23419360.

23. Shang C, Yu J, Zou S, Li H, Cao B, network CA-C. Functional evaluation of TMEM176B and its predictive role for severe respiratory viral infection through integrated analysis of single-cell and bulk RNA-sequencing. J Med Virol. 2024;96(10):e29954. doi: 10.1002/jmv.29954. PMID: 39377494.

24. Dorajoo R, Ali Y, Tay VSY, Kang J, Samydurai S, Liu J, et al. Single-cell transcriptomics of East-Asian pancreatic islets cells. Sci Rep. 2017;7(1):5024. doi: 10.1038/s41598-017-05266-4. PMID: 28694456.

25. van Gurp L, Fodoulian L, Oropeza D, Furuyama K, Bru-Tari E, Vu AN, et al. Generation of human islet cell type-specific identity genesets. Nat Commun. 2022;13(1):2020. doi: 10.1038/s41467-022-29588-8. PMID: 35440614.

26. Wang W, Ledee D. ACAA2 is a ligand-dependent coactivator for thyroid hormone receptor beta1. Biochem Biophys Res Commun. 2021;576:15-21. doi: 10.1016/j.bbrc.2021.08.073. PMID: 34474245.

27. Kurokawa Y, Matoba R, Nakamori S, Takemasa I, Nagano H, Dono K, et al. PCR-array gene expression profiling of hepatocellular carcinoma. 2004;23(1):135-42.

28. Fatima SS, Rehman R, Baig M, Khan TA. New roles of the multidimensional adipokine: chemerin. Peptides. 2014;62:15-20. doi: 10.1016/j.peptides.2014.09.019. PMID: 25278490.

29. Huang HW, Liang BY, Li YX. Association of Polymorphisms in STRA6 and RARRES2 Genes with Type 2 Diabetes in Southern Han Chinese. Biomed Res Int. 2016;2016:6589793. doi: 10.1155/2016/6589793. PMID: 27446956.

30. Dou J, Liu P, Wang J, Zhang X. Effect of hepatitis C virus core shadow protein expressed in human hepatoma cell line on human gene expression profiles. J Gastroenterol Hepatol. 2006;21(12):1794-800. doi: 10.1111/j.1440-1746.2006.04380.x. PMID: 17074016.

31. Makhammajanov Z, Kabayeva A, Auganova D, Tarlykov P, Bukasov R, Turebekov D, et al. Candidate protein biomarkers in chronic kidney disease: a proteomics study. Sci Rep. 2024;14(1):14014. doi: 10.1038/s41598-024-64833-8. PMID: 38890379.

32. Shuwen H, Xi Y, Qing Z, Jing Z, Wei WJCM. Predicting biomarkers from classifier for liver metastasis of colorectal adenocarcinomas using machine learning models. 2020;9(18):6667-78. doi: 10.1002/cam4.3289.

33. Yamaji K, Iwabuchi S, Tokunaga Y, Hashimoto S, Yamane D, Toyama S, et al. Molecular insights of a CBP/beta-catenin-signaling inhibitor on nonalcoholic steatohepatitis-induced liver fibrosis and disorder. Biomed Pharmacother. 2023;166:115379. doi: 10.1016/j.biopha.2023.115379. PMID: 37647690.

34. Wu L, Li Q, Lu F, Qian L, Pan Y, Chen C, et al. Mitochondrial pyruvate carrier 2 mitigates acute kidney injury via sustaining mitochondrial metabolism. Int J Biol Sci. 2024;20(11):4551-65. doi: 10.7150/ijbs.98627. PMID: 39247825.

35. Wyler E, Mosbauer K, Franke V, Diag A, Gottula LT, Arsie R, et al. Transcriptomic profiling of SARS-CoV-2 infected human cell lines identifies HSP90 as target for COVID-19 therapy. iScience. 2021;24(3):102151. doi: 10.1016/j.isci.2021.102151. PMID: 33585804.

36. Vastrad BM, Vastrad CM. Bioinformatics analysis of expression profiling by high throughput sequencing for identification of potential key genes among SARS-CoV-2/COVID 19. 2021. doi: 10.21203/rs.3.rs-122015/v2.

37. Gallo G, Forte M, Cotugno M, Marchitti S, Stanzione R, Tocci G, et al. Polymorphic variants at NDUFC2, encoding a mitochondrial complex I subunit, associate with cardiac hypertrophy in human hypertension. Mol Med. 2023;29(1):107. doi: 10.1186/s10020-023-00701-x. PMID: 37558995.

38. Tan Y, Ma Y, Guo S, Lin Y. Association of abnormal NDUFB2 and UQCRH expression with venous thromboembolism in patients with liver cirrhosis. Medicine (Baltimore). 2024;103(1):e36868. doi: 10.1097/MD.0000000000036868. PMID: 38181234.

39. Liu B, Yi W, Mao X, Yang L, Rao C. Enoyl coenzyme A hydratase 1 alleviates nonalcoholic steatohepatitis in mice by suppressing hepatic ferroptosis. Am J Physiol Endocrinol Metab. 2021;320(5):E925-E37. doi: 10.1152/ajpendo.00614.2020. PMID: 33813878.

40. Shen J, Zhu Y, Zhang S, Lyu S, Lyu C, Feng Z, et al. Vitronectin-activated alphavbeta3 and alphavbeta5 integrin signalling specifies haematopoietic fate in human pluripotent stem cells. Cell Prolif. 2021;54(4):e13012. doi: 10.1111/cpr.13012. PMID: 33656760.

41. Bera A, Subramanian M, Karaian J, Eklund M, Radhakrishnan S, Gana N, et al. Functional role of vitronectin in breast cancer. PLoS One. 2020;15(11):e0242141. doi: 10.1371/journal.pone.0242141. PMID: 33211735.

42. Dat le T, Matsuo T, Yoshimaru T, Kakiuchi S, Goto H, Hanibuchi M, et al. Identification of genes potentially involved in bone metastasis by genome-wide gene expression profile analysis of non-small cell lung cancer in mice. Int J Oncol. 2012;40(5):1455-69. doi: 10.3892/ijo.2012.1348. PMID: 22294041.

43. Feng Y, Zhou YH, Zhao J, Su XL, Chen NX, Zhao YQ, et al. Prognostic biomarker GSTK1 in head and neck squamous cell carcinoma and its correlation with immune infiltration and DNA methylation. Front Genet. 2023;14:1041042. doi: 10.3389/fgene.2023.1041042. PMID: 36936420.

44. Lv L, Ren S, Jiang H, Yan R, Chen W, Yan R, et al. The oral administration of Lacticaseibacillus casei Shirota alleviates acetaminophen-induced liver injury through accelerated acetaminophen metabolism via the liver-gut axis in mice. mSphere. 2024;9(1):e0067223. doi: 10.1128/msphere.00672-23. PMID: 38193757.

45. Ohrnberger S, Thavamani A, Braeuning A, Lipka DB, Kirilov M, Geffers R, et al. Dysregulated serum response factor triggers formation of hepatocellular carcinoma. Hepatology. 2015;61(3):979-89. doi: 10.1002/hep.27539. PMID: 25266280.

46. Pu Y, Yang J, Pan Q, Li C, Wang L, Xie X, et al. MGST3 regulates BACE1 protein translation and amyloidogenesis by controlling the RGS4-mediated AKT signaling pathway. J Biol Chem. 2024;300(8):107530. doi: 10.1016/j.jbc.2024.107530. PMID: 38971310.

47. Sun J, Wang J, Zhang N, Yang R, Chen K, Kong D. Identification of global mRNA expression profiles and comprehensive bioinformatic analyses of abnormally expressed genes in cholestatic liver disease. Gene. 2019;707:9-21. doi: 10.1016/j.gene.2019.04.078. PMID: 31048068.

48. Song M-K, Cho Y, Jeong S-C, Ryu J-CJT, Sciences EH. Analysis of gene expression changes in relation to hepatotoxicity induced by perfluorinated chemicals in a human hepatoma cell line. 2016;8:114-27. doi: 10.1007/s13530-016-0269-x.

49. Luo P, Yan H, Du J, Chen X, Shao J, Zhang Y, et al. PLK1 (polo like kinase 1)-dependent autophagy facilitates gefitinib-induced hepatotoxicity by degrading COX6A1 (cytochrome c oxidase subunit 6A1). Autophagy. 2021;17(10):3221-37. doi: 10.1080/15548627.2020.1851492. PMID: 33315519.

50. Liu D, Fan J, Zeng W, Zhou Y, Ingvarsson S, Chen H. Quantitative analysis of miRNA expression in several developmental stages of human livers. Hepatol Res. 2010;40(8):813-22. doi: 10.1111/j.1872-034X.2010.00683.x. PMID: 20649821.

51. Sun X, Zhang W, Ramdas L, Stivers DN, Jones DM, Kantarjian HM, et al. Comparative analysis of genes regulated in acute myelomonocytic leukemia with and without inv(16)(p13q22) using microarray techniques, real-time PCR, immunohistochemistry, and flow cytometry immunophenotyping. Mod Pathol. 2007;20(8):811-20. doi: 10.1038/modpathol.3800829. PMID: 17571080.

52. Liao J, Zhang Y, Chen X, Zhang J. The Roles of Peroxiredoxin 6 in Brain Diseases. Mol Neurobiol. 2021;58(9):4348-64. doi: 10.1007/s12035-021-02427-5. PMID: 34013449.

53. Pollin G, Mathison AJ, de Assuncao TM, Thomas A, Zeighami A, Salmonson A, et al. Ehmt2 inactivation in pancreatic epithelial cells shapes the transcriptional landscape and inflammation response of the whole pancreas. Front Genet. 2024;15:1412767. doi: 10.3389/fgene.2024.1412767. PMID: 38948355.

54. Wang QC, Zheng Q, Tan H, Zhang B, Li X, Yang Y, et al. TMCO1 Is an ER Ca(2+) Load-Activated Ca(2+) Channel. Cell. 2016;165(6):1454-66. doi: 10.1016/j.cell.2016.04.051. PMID: 27212239.

55. Chu W, Guan L, Huang D, Ren Y, Zhou Y. Lovastatin exerts protective effects on endothelial cells via upregulation of PTK2B. Exp Ther Med. 2016;12(3):1741-9. doi: 10.3892/etm.2016.3547. PMID: 27602089.

56. Di Marco T, Bianchi F, Sfondrini L, Todoerti K, Bongarzone I, Maffioli EM, et al. COPZ1 depletion in thyroid tumor cells triggers type I IFN response and immunogenic cell death. Cancer Lett. 2020;476:106-19. doi: 10.1016/j.canlet.2020.02.011. PMID: 32061953.

57. Richards VE, Chau B, White MR, McQueen CA. Hepatic gene expression and lipid homeostasis in C57BL/6 mice exposed to hydrazine or acetylhydrazine. Toxicol Sci. 2004;82(1):318-32. doi: 10.1093/toxsci/kfh232. PMID: 15282401.

58. Condamine T, Le Texier L, Howie D, Lavault A, Hill M, Halary F, et al. Tmem176B and Tmem176A are associated with the immature state of dendritic cells. J Leukoc Biol. 2010;88(3):507-15. doi: 10.1189/jlb.1109738. PMID: 20501748.

59. Yu CC, Lo WL, Chen YW, Huang PI, Hsu HS, Tseng LM, et al. Bmi-1 Regulates Snail Expression and Promotes Metastasis Ability in Head and Neck Squamous Cancer-Derived ALDH1 Positive Cells. J Oncol. 2011;2011. doi: 10.1155/2011/609259. PMID: 20936121.

60. Hallmann K, Kudin AP, Zsurka G, Kornblum C, Reimann J, Stuve B, et al. Loss of the smallest subunit of cytochrome c oxidase, COX8A, causes Leigh-like syndrome and epilepsy. Brain. 2016;139(Pt 2):338-45. doi: 10.1093/brain/awv357. PMID: 26685157.

61. Zabala V, Boylan JM, Thevenot P, Frank A, Senthoor D, Iyengar V, et al. Transcriptional changes during hepatic ischemia-reperfusion in the rat. PLoS One. 2019;14(12):e0227038. doi: 10.1371/journal.pone.0227038. PMID: 31891620.

62. Jozsi M, Barlow PN, Meri S. Editorial: Function and Dysfunction of Complement Factor H. Front Immunol. 2021;12:831044. doi: 10.3389/fimmu.2021.831044. PMID: 35095933.

63. Shajari N, Ramezani A, Tahmasebi A, Anbardar MH, Roshanizadeh Z, Ghaderi AJMEJoC. Feature Selection Algorithms Combined with Experimental Validation Reveal COL10A1 and TMPRSS4 Genes as Potential Diagnostic Biomarkers in Pancreatic Ductal Adenocarcinoma. 2024. doi: 10.30476/mejc.2024.102622.2100.

64. Wan Z, Gu J, Balaji U, Bojmar L, Molina H, Heissel S, et al. Optimization of ultracentrifugation-based method to enhance the purity and proteomic profiling depth of plasma-derived extracellular vesicles and particles. J Extracell Biol. 2024;3(7):e167. doi: 10.1002/jex2.167. PMID: 39045341.

65. Dahabieh MS, Di Pietro E, Jangal M, Goncalves C, Witcher M, Braverman NE, et al. Peroxisomes and cancer: The role of a metabolic specialist in a disease of aberrant metabolism. Biochim Biophys Acta Rev Cancer. 2018;1870(1):103-21. doi: 10.1016/j.bbcan.2018.07.004. PMID: 30012421.

66. Zhengqi Q, Zezhi G, Lei J, He Q, Jinyao P, Ying A. Prognostic role of PHYH for overall survival (OS) in clear cell renal cell carcinoma (ccRCC). Eur J Med Res. 2021;26(1):9. doi: 10.1186/s40001-021-00482-1. PMID: 33468235.

67. Popovici V, Budinska E, Tejpar S, Weinrich S, Estrella H, Hodgson G, et al. Identification of a poor-prognosis BRAF-mutant-like population of patients with colon cancer. J Clin Oncol. 2012;30(12):1288-95. doi: 10.1200/JCO.2011.39.5814. PMID: 22393095.

68. Zhu Y, Zhang G, Lin S, Shi J, Zhang H, Hu J. Sec61beta facilitates the maintenance of endoplasmic reticulum homeostasis by associating microtubules. Protein Cell. 2018;9(7):616-28. doi: 10.1007/s13238-017-0492-5. PMID: 29168059.

69. Lang S, Pfeffer S, Lee PH, Cavalie A, Helms V, Forster F, et al. An Update on Sec61 Channel Functions, Mechanisms, and Related Diseases. Front Physiol. 2017;8:887. doi: 10.3389/fphys.2017.00887. PMID: 29163222.

70. Ojha NK, Lole KS. Hepatitis E virus ORF1 encoded non structural protein-host protein interaction network. Virus Res. 2016;213:195-204. doi: 10.1016/j.virusres.2015.12.007. PMID: 26689634.

71. Liu J, Hermo L, Ding D, Wei C, Mann JM, Yan X, et al. SYPL1 defines a vesicular pathway essential for sperm cytoplasmic droplet formation and male fertility. Nat Commun. 2023;14(1):5113. doi: 10.1038/s41467-023-40862-1. PMID: 37607933.

72. Yao J, Cheng Y, Zhang D, Fan J, Zhao Z, Li Y, et al. Identification of key genes, MicroRNAs and potentially regulated pathways in alcoholic hepatitis by integrative analysis. Gene. 2019;720:144035. doi: 10.1016/j.gene.2019.144035. PMID: 31404595.

73. Lindquist JA, Brandt S, Bernhardt A, Zhu C, Mertens PR. The role of cold shock domain proteins in inflammatory diseases. J Mol Med (Berl). 2014;92(3):207-16. doi: 10.1007/s00109-014-1136-3. PMID: 24562821.

74. Hou H, Wang F, Zhang W, Wang D, Li X, Bartlam M, et al. Structure-functional analyses of CRHSP-24 plasticity and dynamics in oxidative stress response. J Biol Chem. 2011;286(11):9623-35. doi: 10.1074/jbc.M110.177436. PMID: 21177848.

75. Alshabi AM, Shaikh IA, Vastrad BM, Vastrad CM. Identification of differentially expressed genes and enriched pathways in SARS-CoV-2/COVID-19 using bioinformatics analysis. 2020. doi: 10.21203/rs.3.rs-122015/v1.

76. Jun MH, Jun YW, Kim KH, Lee JA, Jang DJ. Characterization of the cellular localization of C4orf34 as a novel endoplasmic reticulum resident protein. BMB Rep. 2014;47(10):563-8. doi: 10.5483/bmbrep.2014.47.10.252. PMID: 24499674.

77. Warner N, Burberry A, Pliakas M, McDonald C, Nunez G. A genome-wide small interfering RNA (siRNA) screen reveals nuclear factor-kappaB (NF-kappaB)-independent regulators of NOD2-induced interleukin-8 (IL-8) secretion. J Biol Chem. 2014;289(41):28213-24. doi: 10.1074/jbc.M114.574756. PMID: 25170077.

78. Cai C, Lin J, Li J, Wang X-D, Xu L-M, Chen D-Z, et al. miRNA‐432 and SLC38A1 as Predictors of Hepatocellular Carcinoma Complicated with Alcoholic Steatohepatitis. 2022;2022(1):4832611. doi: 10.1155/2022/4832611.

79. Yuan L, Chen L, Qian K, Wang G, Lu M, Qian G, et al. A novel correlation between ATP5A1 gene expression and progression of human clear cell renal cell carcinoma identified by co‑expression analysis. Oncol Rep. 2018;39(2):525-36. doi: 10.3892/or.2017.6132. PMID: 29207195.

80. Protiva P, Gong J, Sreekumar B, Torres R, Zhang X, Belinsky GS, et al. Pigment Epithelium-Derived Factor (PEDF) Inhibits Wnt/beta-catenin Signaling in the Liver. Cell Mol Gastroenterol Hepatol. 2015;1(5):535-49 e14. doi: 10.1016/j.jcmgh.2015.06.006. PMID: 26473164.

81. Sinha NK, Ordureau A, Best K, Saba JA, Zinshteyn B, Sundaramoorthy E, et al. EDF1 coordinates cellular responses to ribosome collisions. Elife. 2020;9. doi: 10.7554/eLife.58828. PMID: 32744497.

82. Oittinen M, Popp A, Kurppa K, Lindfors K, Maki M, Kaikkonen MU, et al. Polycomb Repressive Complex 2 Enacts Wnt Signaling in Intestinal Homeostasis and Contributes to the Instigation of Stemness in Diseases Entailing Epithelial Hyperplasia or Neoplasia. Stem Cells. 2017;35(2):445-57. doi: 10.1002/stem.2479. PMID: 27570105.
