# Supplementary material for: Multiomics in silico analysis identifies TM4SF4 as a cell surface target in hepatocellular carcinoma
Source: PLoS One. 2025 Feb 25;20(2):e0307048. doi: 10.1371/journal.pone.0307048 (PMC11856526; doi:10.1371/journal.pone.0307048)
Supplement: Attachment — Submitted filename: Response to Reviewers.docx [file pone.0307048.s010.docx]

**Academic Editor**

**The conclusion presented at the end of the abstract of manuscript should be carefully refined to ensure consistency with the final conclusion of the manuscript.**

Thank you for the comments. The conclusion at the end of the Abstract has been revised as follows:

**********

Taken together, TM4SF4 is proposed as a promising cell surface target in HCC due to its high expression in HCC cells with restricted expression profile in non-cancerous tissues, and association with HCC oncogenic pathways.

**********

**In particular, it is important to address whether the identification of both TM4SF4+ and TM4SF4- cell populations across various normal human tissues impacts the validity of using TM4SF4 as a cell surface target for hepatocellular carcinoma treatment. Does the presence of TM4SF4 in non-cancerous tissues undermine its potential as a specific therapeutic target for hepatocellular carcinoma? This key consideration should be thoroughly discussed to ensure the conclusion reflects the broader implications of the findings.**

Thank you for the comments. The issues raised (*i.e.,* whether TM4SF4 expression in non-cancerous tissues undermine its therapeutic potential) have now been addressed in the limitations section of the Discussion as follows (pages 26-27):

**********

The limitations of this study are acknowledged as follows: 1) TM4SF4 is present in normal bile ducts (*i.e.,* cholangiocytes) and the gallbladder epithelial cells where they are in contact with the adjacent stroma containing immune cells and lymphocytes. Hence, anti-TM4SF4 immunotherapy may cause toxicity to these sites. However, immune-related injury of bile ducts after immune-checkpoint inhibitor treatment can be treated with immunosuppressive agents such as prednisolone or mycophenolate mofetil [129, 130]; 2) Targeting TM4SF4 systematically can cause toxicity to pancreatic α cells that express TM4SF4 [131, 132]. However, our findings have demonstrated that TM4SF4 expression is more restricted compared with other more commonly investigated HCC surface therapeutic targets (*e.g.*, GPC3 and CD147) which are highly expressed in tissues critical for survival such as the lungs and brain. Essentially, the localization of TM4SF4 in the bile duct canaliculi of the hepatocytes where immune cells are unable to access, suggests a lower likelihood of off-tumor effects when anti-TM4SF4 therapies are locally administered near HCC tumor sites. **********

The Conclusion section of the manuscript has also been revised as follows (page 27):

**********

This study provides evidence to support the development of anti-TM4SF4 immunotherapies such as CAR T cells against HCCs. The more restricted expression profile of TM4SF4 in normal human tissues versus other common therapeutic targets in HCC further supports the use of anti-TM4SF4 targeted agents against the disease. Moreover, the association of *TM4SF4* expression with mitochondrial processes and energy production GOs in scRNA-seq dataset of HCC cells are reported for the first time. Finally, the shortlisting and ranking methodologies proposed in this study can be implemented independently to uncover the optimal cell surface targets for therapeutic development in other malignancies.

**********

References

129.	Haanen J, Carbonnel F, Robert C, Kerr KM, Peters S, Larkin J, et al. Management of toxicities from immunotherapy: ESMO Clinical Practice Guidelines for diagnosis, treatment and follow-up. Ann Oncol. 2017;28(suppl_4):iv119-iv42. doi: 10.1093/annonc/mdx225. PMID: 28881921.

130.	Murayama A, Tajiri K, Nakaya A, Ito H, Hayashi Y, Entani T, et al. Intrahepatic Bile Duct Injury as a Hepatic Immune-Related Adverse Event after Immune-Checkpoint Inhibitor Treatment. Case Rep Gastroenterol. 2021;15(2):645-51. doi: 10.1159/000516199. PMID: 34616270.

131.	Wendt A, Eliasson L. Pancreatic alpha-cells - The unsung heroes in islet function. Semin Cell Dev Biol. 2020;103:41-50. doi: 10.1016/j.semcdb.2020.01.006. PMID: 31983511.

132.	Roder PV, Wu B, Liu Y, Han W. Pancreatic regulation of glucose homeostasis. Exp Mol Med. 2016;48(3):e219. doi: 10.1038/emm.2016.6. PMID: 26964835.

**Journal’s Editorial**

**1. Please ensure that your manuscript meets PLOS ONE's style requirements, including those for file naming. The PLOS ONE style templates can be found at**

[**https://journals.plos.org/plosone/s/file?id=wjVg/PLOSOne_formatting_sample_main_body.pdf**](https://journals.plos.org/plosone/s/file?id=wjVg/PLOSOne_formatting_sample_main_body.pdf) **and**

[**https://journals.plos.org/plosone/s/file?id=ba62/PLOSOne_formatting_sample_title_authors_affiliations.pdf**](https://journals.plos.org/plosone/s/file?id=ba62/PLOSOne_formatting_sample_title_authors_affiliations.pdf)

Thank you for the comments. We have implemented all the requirements as necessary.

**2. Thank you for stating the following financial disclosure:**

**This work was supported by the Fundamental Research Grant Scheme (FRGS) (grant no: 203.PPSP.6171388) awarded to KKW by the Ministry of Higher Education (MoHE) Malaysia.”**

**Please state what role the funders took in the study. If the funders had no role, please state: "The funders had no role in study design, data collection and analysis, decision to publish, or preparation of the manuscript."**

**If this statement is not correct you must amend it as needed.**

**Please include this amended Role of Funder statement in your cover letter; we will change the online submission form on your behalf.**

Thank you for the comments. The funder had no role in the study apart from providing the research funding, thus the statement “The funders had no role in study design, data collection and analysis, decision to publish, or preparation of the manuscript.” is correct.

**3. Please review your reference list to ensure that it is complete and correct. If you have cited papers that have been retracted, please include the rationale for doing so in the manuscript text, or remove these references and replace them with relevant current references. Any changes to the reference list should be mentioned in the rebuttal letter that accompanies your revised manuscript. If you need to cite a retracted article, indicate the article’s retracted status in the References list and also include a citation and full reference for the retraction notice.**

Thank you for the comments. The reference list has been checked and it is complete and correct.

**Reviewer #1**

**This paper presents a detailed analysis of TM4SF4, whose expression is increased in HCC, by using various databases. In addition, immunostaining with anti-TM4SF4 antibodies were performed to confirm positive cells in HCC tissues. As noted in the conclusion, TM4SF4-neutralizing antibodies may be a new therapeutic target in HCC. However, the correlation between TM4SF4 overexpression and poor prognosis was low from the KM plot (Fig. S3). This suggests that TM4SF4 is not directly associated with tumor growth, and that genes with correlated high expression or interacting downstream genes or signaling pathway may be most important.**

**(1) Reanalyze the scRNA-seq data in Fig. 9, and check whether the representative genes that correlate with TM4SF4 expression are consistent with the proteins that have been published in Gene Card and other journals.**

**Are these genes related to TM4SF4 associated with HCC malignancy by searching such as TCGA database?**

**I would like to conduct additional analysis of the above, and hope that the impact of TM4SF4 on downstream signals will lead to the meaningful to develop an anticancer drug for targeting TM4SF4.**

Thank you for the comments. For the original version of the manuscript’s Fig. 9 (now designated as Fig. 8 due to the exclusion of a main figure, *i.e.,* the molecular docking figure previously designated as Fig. 8, to address the second comment below), we have conducted literature review on all 32 genes associated with *TM4SF4* expression in HCC but not in NTLs (according to r >0.6 cut-off). There has not been a functional study demonstrating a direct link between TM4SF4 with any of those 32 genes due to the lack of transcriptomics- or proteomics-wide study upon knockdown or overexpression of TM4SF4 expression in HCC cells that may alter the expression of these genes.

Nonetheless, potential associations between TM4SF4 with any of the 32 genes are shown by shared altered expression profile in specific malignancies or conditions. For instance, *SCP2* and *TM4SF4* were part of 719 specific genes associated with liver metastasis of colon carcinoma [81], or *Agt* and *Tm4sf4* were downregulated by tauroursodeoxycholic acid, an anti-hepatocarcinogenesis compound [83], in rat hepatocytes [84].

We have included a new table in Fig. 8 summarizing the potential links of five selected top genes most highly associated with *TM4SF4*, as well as each gene’s normal functions for added contexts. The complete descriptions the normal functions of all 32 genes and their potential links with TM4SF4 are presented in a new S5 Table. We have also labeled the top eight genes (selected the top eight due to space constraint in the figure) within the scatter plot of Fig. 8 for better clarity.

The following descriptions have been added in the Results section (page 20):

**********

The top eight genes with the highest r values with *TM4SF4* expression are shown in the scatter plot (Fig. 8). Five of these top genes (*CLU*, *SCP2*, *AGT*, *ALB*, and *SDHC*) were described in terms of their normal functions and potential association with *TM4SF4*, and such descriptions for all 32 genes are presented in S5 Table.

**********

Regarding TCGA cases, the following descriptions have been added in the Results section (page 21):

**********

In terms of The Cancer Genome Atlas (TCGA) cases, each gene's expression values (transcript per million, TPM) in HCC cases (n=369) vs non-HCC liver cases (n=160) were compared according to the Gene Expression Profiling Interactive Analysis 2 (GEPIA2) database [89]. Six of the 32 genes (*MPC2*, *NDUFC2*, *COX6A1*, *TMCO1*, *COPZ1*, and *COX8A*), as well as *TM4SF4*, showed significantly higher expression values in HCC compared with non-HCC liver cases (all *p*<0.001; S5 Table). The rest of the genes (n=26) did not show significant difference between the two groups, however 22 of these genes showed higher expression values in the HCC group (S5 Table).

**********

Regarding potential TM4SF4 downstream signaling, the following descriptions have been added in the Discussion section (page 25):

**********

Four of the genes that contributed to the enrichment of mitochondrial components (*MPC2*, *NDUFC2*, *COX6A1*, and *COX8A*) were significantly overexpressed in TCGA HCC cases as described previously in S5 Table. As *TM4SF4* was also overexpressed in TCGA HCC cases, this suggests that TM4SF4 signaling may lead to upregulation of any of these four genes’ expression. Although there have not been studies showing the direct association between TM4SF4 with these four genes, co-expression patterns in independent studies may support this observation. In particular, *MPC2* may appear as a stronger candidate among the other genes where the expression of both *MPC2* and *TM4SF4* were suppressed in human malignant epithelial cells infected by SARS-CoV-2 versus controls [125, 126]. However, copy-number variation analyses showed that MPC2 was frequently amplified in a specific molecular subtype of HCC termed as the “mixed group” subtype characterized by higher variability in metabolic activity, compared to the quiescent subtype defined by low metabolic activity and cholesterol subtype characterized by reliance on cholesterol synthesis pathways [127]. Hence, direct mechanistic studies of TM4SF4 regulation of genes expression are required to demonstrate the downstream signaling controlled by TM4SF4 in HCC.

**********

The figure legends of Fig. 8 have also been expanded to include this sentence:

**********

The top eight genes with the highest r values with TM4SF4 expression were labeled in the scatter plot.

**********

**(2) Please explain why the authors showed the result in Fig.8? Although the structure of the inhibitory site of TM4SF4 protein is abruptly introduced in Fig. 8, I do not feel the significance of treating it in the text unless the inhibitors are actually synthesized and tested in mice or other animals.**

Thank you for the comments. The molecular docking analyses were initially included to address the therapeutic aspect of the manuscript. Nonetheless, we have now completely removed the results of this figure (previously designated as Fig. 8), its methods, discussion, and all corresponding texts from the manuscript to maintain focus on the core findings of the study. We apologize for any confusion caused.

References

81.	Liu J, Wang D, Zhang C, Zhang Z, Chen X, Lian J, et al. Identification of liver metastasis-associated genes in human colon carcinoma by mRNA profiling. Chin J Cancer Res. 2018;30(6):633-46. doi: 10.21147/j.issn.1000-9604.2018.06.08. PMID: 30700932.

83.	Vandewynckel YP, Laukens D, Devisscher L, Paridaens A, Bogaerts E, Verhelst X, et al. Tauroursodeoxycholic acid dampens oncogenic apoptosis induced by endoplasmic reticulum stress during hepatocarcinogen exposure. Oncotarget. 2015;6(29):28011-25. doi: 10.18632/oncotarget.4377. PMID: 26293671.

84.	Castro RE, Sola S, Ma X, Ramalho RM, Kren BT, Steer CJ, et al. A distinct microarray gene expression profile in primary rat hepatocytes incubated with ursodeoxycholic acid. J Hepatol. 2005;42(6):897-906. doi: 10.1016/j.jhep.2005.01.026. PMID: 15885361.

89.	Tang Z, Kang B, Li C, Chen T, Zhang Z. GEPIA2: an enhanced web server for large-scale expression profiling and interactive analysis. Nucleic Acids Res. 2019;47(W1):W556-W60. doi: 10.1093/nar/gkz430. PMID: 31114875.

125.	Wyler E, Mosbauer K, Franke V, Diag A, Gottula LT, Arsie R, et al. Transcriptomic profiling of SARS-CoV-2 infected human cell lines identifies HSP90 as target for COVID-19 therapy. iScience. 2021;24(3):102151. doi: 10.1016/j.isci.2021.102151. PMID: 33585804.

126.	Vastrad BM, Vastrad CM. Bioinformatics analysis of expression profiling by high throughput sequencing for identification of potential key genes among SARS-CoV-2/COVID 19. 2021. doi: 10.21203/rs.3.rs-122015/v2.

127.	Deng W, Zhu P, Xu H, Hou X, Chen W. Classification and Prognostic Characteristics of Hepatocellular Carcinoma Based on Glycolysis Cholesterol Synthesis Axis. J Oncol. 2022;2022:2014625. doi: 10.1155/2022/2014625. PMID: 36213830.

**Reviewer #2**

**The authors of this article have applied a multi-omics data analysis of TM4SF4 as a cell surface target in hepatocellular carcinoma. This title is worthy of investigation and it is evident that lots of effort was put into writing this informative manuscript. However, some comments are mentioned below that need more clarification, explanation, or modification.**

**1. Please include the type of study in the title.**

Thank you for the comment. The title has been revised to state the type of study as follows: **********

Multiomics *in silico* analysis identifies TM4SF4 as a cell surface target in hepatocellular carcinoma

**********

**2. The abstract of the article is better to be structured.**

Thank you for the comment. The Abstract has been revised to be more structured while adhering to PLOS One's Abstract format as follows:

**********

The clinical application of cellular immunotherapy in hepatocellular carcinoma (HCC) is impeded by the lack of a cell surface target frequently expressed in HCC cells and with minimal presence in normal tissues to reduce on-target, off-tumor toxicity. To address this, an *in silico* multomics analysis was conducted to identify an optimal therapeutic target in HCC. A longlist of genes (n=12,948) expressed in HCCs according to The Human Protein Atlas database were examined. Eight genes were shortlisted to identify one with the highest expression in HCCs, without being shed into circulation, and with restrictive expression profile in other normal human tissues. A total of eight genes were shortlisted and subsequently ranked according to the combination of their transcript and protein expression levels in HCC cases (n=791) derived from four independent datasets. TM4SF4 was the top-ranked target with the highest expression in HCCs. TM4SF4 showed more favorable expression profile with significantly lower expression in normal human tissues but more highly expressed in HCC compared with seven other common HCC therapeutic targets. Furthermore, scRNA-seq and immunohistochemistry datasets showed that TM4SF4 was absent in immune cell populations but highly expressed in the bile duct canaliculi of hepatocytes, regions inaccessible to immune cells. In scRNA-seq dataset of HCCs, *TM4SF4* expression was positively associated with mitochondrial components and oxidative phosphorylation Gene Ontologies in HCC cells (n=15,787 cells), suggesting its potential roles in mitochondrial-mediated oncogenic effects in HCC. Taken together, TM4SF4 is proposed as a promising cell surface target in HCC due to its high expression in HCC cells with restricted expression profile in non-cancerous tissues, and association with HCC oncogenic pathways.

**********

**3. The third paragraph of the introduction section needs to be concise. The amount of details that were provided may distract the attention of the reader from the main purpose of the study.**

Thank you for the comments. The third paragraph of the Introduction section has been revised to be concise as follows (pages 3-4):

**********

Immunotherapies such as chimeric antigen receptor (CAR) T-cell therapy are under investigation for HCC. However, their clinical translation is impeded by the heterogeneity of cell surface protein expression and off-tumor toxicities due to target expression in normal tissues [12]. One example is glypican-3 (GPC3; a heparan sulfate proteoglycan), where its expression is low in normal liver tissues but expressed in around 70% of HCC patients [13, 14]. However, GPC3 is expressed in multiple normal tissues including the lungs, mesothelium, mammary epithelium, ovaries and the brain [15-17]. Therapeutic T cells targeting antigen expressed in normal critical tissues including the brain and lung tissues often result in toxicities [18-20]. Furthermore, serum GPC3 is commonly increased in HCC due to cell surface GPC3 shedding, and shed GPC3 (sGPC3) is a negative regulator of cell surface GPC3 leading to inhibition of HCC cell growth [21, 22], and blocking anti-GPC3 CAR T cells binding, reducing treatment efficacy [23].

**********

**4. The authors have discussed some methods of the study in the last paragraph of the introduction section. Let them just remain in their appropriate place, i.e. the method section. You should include two things at the end of the introduction section: 1. the objective(s) of the study and 2. the novelty of the study**

Thank you for the comments. The last paragraph of the Introduction section has been revised as follows (page 4):

**********

In this *in silico* study, an exhaustive exploration for the optimal cell surface target highly expressed in HCC but absent from majority of normal human tissues was conducted according to the combination of multiple publicly available transcriptomics and proteomics datasets. Potential therapeutic targets were objectively shortlisted, and TM4SF4 was identified as the most promising candidate. By leveraging multiple independent datasets, as well as comparison with GPC3 and other common surface targets studied in HCC, the therapeutic potential of TM4SF4 was established. Furthermore, the potential functional roles of TM4SF4 in HCC were explored, further supporting its future therapeutic development against the deadly malignancy.

**********

**5. Please mention the type of the study in the method section.**

Thank you for the comment. The following sentence has been added in the Materials and Methods section (page 4):

**********

This is an *in silico* study utilizing publicly available multiomics datasets, to identify and characterize potential cell surface therapeutic targets in HCC.

**********

**6. Have you utilized any control group or not?**

Thank you for the comment. We have added the following descriptions in the Materials and Methods section (page 10):

**********

Control groups were used extensively in this study. Normal human tissues as controls were included in the comparisons during target shortlisting and validation. These included normal tissues datasets from the FANTOM5 (n=45), GTEx (n=54), and the HPA (n=253). Moreover, non-cancerous human tissues from the HPA immunohistochemistry (IHC) datasets (*e.g.*, gallbladder, duodenum, small intestine, stomach, pancreas, colon) were used as controls for comparison with HCC tissues in terms of TM4SF4 protein expression patterns, as detailed in subsequent sections. Additionally, NTL single cells were used as controls for intra-tumoral comparisons to identify genes specifically correlated with TM4SF4 expression in HCC but not NTL cells, in single-cell RNA-seq (scRNA-seq) analysis as detailed in subsequent sections.

**********

**7. Have you provided any ethics approval number from a recognized institution? If yes, please mention it.**

Thank you for the comments. We have added the following descriptions in the Materials and Methods section (page 6):

**********

This *in silico* study did not involve the direct collection of data from human participants, nor did it utilize human specimens or tissues in a manner requiring ethical approval. All data used in this study were obtained from publicly available sources and anonymized datasets. Therefore, additional review and approval by an institutional review board or ethics committee was not required for this study.

**********

**8. It is recommended to transfer all the website links to the references section.**

Thank you for the comment. We have excluded the website links from the main text, as the original publications for each dataset are already cited and also to comply with the journal’s referencing format. Here are the revised sentences in the Materials and Methods section after exclusion of the corresponding website links (pages 5-6):

**********

The Chinese Human Proteome Project (CNHPP) RNA-seq dataset of 35 fresh paired tissues *i.e.,* 35 adjacent non-tumor liver (NTL) cells and 35 HCC cases were retrieved from the supplementary information of its publication [39]. An independent transcriptomics dataset (microarray) of HCC (n=225) and paired NTL (n=220) were obtained from the Gene Expression Omnibus database (ID: GSE14520) [40, 41]. For proteomics dataset, the Chinese HCC (CHCC) dataset of 166 paired tissues *i.e.,* 166 HCC and 166 NTL cases whose proteins were measured by mass spectrometry (MS) were retrieved from the Proteomic Data Commons database (ID: PDC000198) [42].

**********

**9. Please mention the inclusion and exclusion criteria for the cases in your study.**

Thank you for the comment. The following inclusion and exclusion criteria have been added in the Materials and Methods section (pages 4-5):

**********

Inclusion criteria for datasets used in this study were: 1) Transcriptomics and proteomics datasets with open access to raw and processed data; 2) Datasets that specifically include HCC and matched control NTL group to allow for paired analysis; 3) Datasets with a sample size of at least 30 per group for quantitative comparisons; 4) Proteomics datasets utilizing mass spectrometry or similar high-throughput methods to quantify protein levels; 5) Datasets with sufficient metadata, including clear labeling of tumor and non-tumor cases, and clinical or demographic data where available.

Exclusion criteria for datasets used in this study were: 1) Datasets not related to gene or protein expression, such as genomic mutation-only studies; 2) Datasets without clear annotations of tumor or non-tumor samples; 3) Studies focusing on other liver diseases (e.g., hepatitis, cirrhosis) without sufficient HCC cases; 4) Datasets containing metastatic or recurrent HCC samples only without sufficient primary HCC cases; 5) Datasets with incomplete metadata (*e.g.*, missing labels of tumor or non-tumor cases) or lacking information about experimental protocols (*e.g.*, the specific platform used in their high-throughput analysis).

**********

**10. Please mention the variables that were investigated in the methods section.**

Thank you for the comment. The following descriptions have been added in the Materials and Methods section (page 13):

**********

The variables investigated in this study, across multiple transcriptomics and proteomics datasets, included: 1) Gene and protein expression levels in HCC vs NTL and other normal tissues (such as the brain and lung tissues); 2) Expression levels of TM4SF4 vs other common cell surface markers studied in HCC; 3) Correlation analyses (Pearson’s r values) to shortlist for genes highly correlated with TM4SF4 in HCC but not NTL cells; 4) Functional annotations of these TM4SF4-correlated genes in HCC via ontologies enrichment analysis to uncover TM4SF4-specific processes in HCCs.

**********

**11. Please improve the cohesion and coherence of the discussion section and make it concise. It does not needed to report the findings of every previous study in a separate sentence. For instance, it is better to combine the findings of several similar previous studies and present them in one informative sentence in order to avoid redundancy.**

Thank you for the comments. Multiple paragraphs of the Discussion section have been shortened by combining findings of several similar previous studies as follows:

Third paragraph of the Discussion section (pages 23-24):

**********

TM4SF4 protein was specifically localized on all sides of the plasma membrane domains of HCCs but only present in the bile duct canaliculi of normal liver tissues, consistent with the IHC data by HPA as reported in this study. Knockdown of TM4SF4 in HCC cells impaired growth potential *in vitro* and repressed tumor growth in a mouse xenograft model of human HCC, [103] similar with another study showing attenuated proliferation and migration of TM4SF4-silenced HCC cells [104]. Collectively, these studies indicate that TM4SF4 functions as an oncoprotein in HCCs.

**********

Fourth paragraph of the Discussion section (page 24):

**********

Accumulating evidence has also established the roles of TM4SF4 as an oncoprotein in other solid tumors. In colorectal cancer (CRC), higher TM4SF4 expression is associated with liver metastasis, worse survival, tumor development and epithelial-mesenchymal transition (EMT) program regulated by TGFβ/Snail, TNFα/NFκB, and thymidylate synthase pathways [105-107]. In lung cancer, TM4SF4 expression is upregulated where it promotes tumor growth, migration, and invasion through activation of PI3K/AKT and JAK2/STAT3 pathways, as well as preserving cancer stem cell features [108-111]. TM4SF4 is also overexpressed in pancreatic cancers as a hub gene and upregulated in chemoresistant ovarian cancer cells [112, 113]. Taken together, TM4SF4 exerts oncogenic effects in multiple types of solid tumors, supporting its potential as a therapeutic target beyond HCC.

**********

Sixth paragraph of the Discussion section (pages 24-25):

**********

In this study, *TM4SF4* expression was positively associated with mitochondrial components, oxidative phosphorylation, and ATP synthesis GOs. Mitochondria, often found in close proximity to the PM for Ca^2+^ signaling or to provide ATP, play key roles in liver metabolism and lipid hormone responses [120-122]. In HCC, the expression of mitochondrial citrate transporter and PM citrate transporter is elevated, and inhibitors targeting these transporters induce apoptosis of HCC cells [123]. More importantly, TM4SF4 overexpression accelerates carbon tetrachloride (CCl_4_)-mediated liver injury, and CCl_4_-mediated liver injury is caused by mitochondrial permeability alterations [124]. Collectively, as TM4SF4 is found on the PM of HCCs, the potential interaction of TM4SF4 with mitochondria in HCC cells may lead to oncogenic effects such as mitochondrial abnormalities, but this proposal remains to be validated experimentally.

**********

References

12.	Guo J, Tang Q. Recent updates on chimeric antigen receptor T cell therapy for hepatocellular carcinoma. Cancer Gene Ther. 2021;28(10-11):1075-87. doi: 10.1038/s41417-020-00259-4. PMID: 33500535.

13.	Gao W, Ho M. The role of glypican-3 in regulating Wnt in hepatocellular carcinomas. Cancer Rep. 2011;1(1):14-9. PMID: 22563565.

14.	Huang SL, Wang YM, Wang QY, Feng GG, Wu FQ, Yang LM, et al. Mechanisms and Clinical Trials of Hepatocellular Carcinoma Immunotherapy. Front Genet. 2021;12:691391. doi: 10.3389/fgene.2021.691391. PMID: 34306031.

15.	Luxardi G, Galli A, Forlani S, Lawson K, Maina F, Dono R. Glypicans are differentially expressed during patterning and neurogenesis of early mouse brain. Biochem Biophys Res Commun. 2007;352(1):55-60. doi: 10.1016/j.bbrc.2006.10.185. PMID: 17107664.

16.	Zhou F, Shang W, Yu X, Tian J. Glypican-3: A promising biomarker for hepatocellular carcinoma diagnosis and treatment. Med Res Rev. 2018;38(2):741-67. doi: 10.1002/med.21455. PMID: 28621802.

17.	Herb BR, Glover HJ, Bhaduri A, Casella AM, Bale TL, Kriegstein AR, et al. Preprint: Single-cell genomics reveals region-specific developmental trajectories underlying neuronal diversity in the prenatal human hypothalamus. bioRxiv. 2021:1-23. doi: 10.1101/2021.07.20.453090.

18.	Thistlethwaite FC, Gilham DE, Guest RD, Rothwell DG, Pillai M, Burt DJ, et al. The clinical efficacy of first-generation carcinoembryonic antigen (CEACAM5)-specific CAR T cells is limited by poor persistence and transient pre-conditioning-dependent respiratory toxicity. Cancer Immunol Immunother. 2017;66(11):1425-36. doi: 10.1007/s00262-017-2034-7. PMID: 28660319.

19.	Morgan RA, Chinnasamy N, Abate-Daga D, Gros A, Robbins PF, Zheng Z, et al. Cancer regression and neurological toxicity following anti-MAGE-A3 TCR gene therapy. J Immunother. 2013;36(2):133-51. doi: 10.1097/CJI.0b013e3182829903. PMID: 23377668.

20.	Brudno JN, Kochenderfer JN. Toxicities of chimeric antigen receptor T cells: recognition and management. Blood. 2016;127(26):3321-30. doi: 10.1182/blood-2016-04-703751. PMID: 27207799.

21.	Zittermann SI, Capurro MI, Shi W, Filmus J. Soluble glypican 3 inhibits the growth of hepatocellular carcinoma in vitro and in vivo. Int J Cancer. 2010;126(6):1291-301. doi: 10.1002/ijc.24941. PMID: 19816934.

22.	Feng M, Kim H, Phung Y, Ho M. Recombinant soluble glypican 3 protein inhibits the growth of hepatocellular carcinoma in vitro. Int J Cancer. 2011;128(9):2246-7. doi: 10.1002/ijc.25549. PMID: 20617511.

23.	Sun L, Gao F, Gao Z, Ao L, Li N, Ma S, et al. Shed antigen-induced blocking effect on CAR-T cells targeting Glypican-3 in Hepatocellular Carcinoma. J Immunother Cancer. 2021;9(4). doi: 10.1136/jitc-2020-001875. PMID: 33833049.

39.	Jiang Y, Sun A, Zhao Y, Ying W, Sun H, Yang X, et al. Proteomics identifies new therapeutic targets of early-stage hepatocellular carcinoma. Nature. 2019;567(7747):257-61. doi: 10.1038/s41586-019-0987-8. PMID: 30814741.

40.	Roessler S, Jia HL, Budhu A, Forgues M, Ye QH, Lee JS, et al. A unique metastasis gene signature enables prediction of tumor relapse in early-stage hepatocellular carcinoma patients. Cancer Res. 2010;70(24):10202-12. doi: 10.1158/0008-5472.CAN-10-2607. PMID: 21159642.

41.	Zhao N, Dang H, Ma L, Martin SP, Forgues M, Ylaya K, et al. Intratumoral gammadelta T-Cell Infiltrates, Chemokine (C-C Motif) Ligand 4/Chemokine (C-C Motif) Ligand 5 Protein Expression and Survival in Patients With Hepatocellular Carcinoma. Hepatology. 2021;73(3):1045-60. doi: 10.1002/hep.31412. PMID: 32502310.

42.	Gao Q, Zhu H, Dong L, Shi W, Chen R, Song Z, et al. Integrated Proteogenomic Characterization of HBV-Related Hepatocellular Carcinoma. Cell. 2019;179(2):561-77 e22. doi: 10.1016/j.cell.2019.08.052. PMID: 31585088.

103.	Wang L, Feng J, Da L, Li Y, Li Z, Zhao M. Adenovirus-mediated delivery of siRNA targeting TM4SF4 attenuated liver cancer cell growth in vitro and in vivo. Acta Biochim Biophys Sin (Shanghai). 2013;45(3):213-9. doi: 10.1093/abbs/gms115. PMID: 23296076.

104.	Mou Y, Ding X. LncRNA ST8SIA6-AS1 facilitates hepatocellular carcinoma progression by governing miR-651-5p/TM4SF4 axis. Anticancer Drugs. 2022;33(8):741-51. doi: 10.1097/CAD.0000000000001326. PMID: 35946523.

105.	Li H, Zhong A, Li S, Meng X, Wang X, Xu F, et al. The integrated pathway of TGFbeta/Snail with TNFalpha/NFkappaB may facilitate the tumor-stroma interaction in the EMT process and colorectal cancer prognosis. Sci Rep. 2017;7(1):4915. doi: 10.1038/s41598-017-05280-6. PMID: 28687755.

106.	Zhang F, Ye J, Guo W, Zhang F, Wang L, Han A. TYMS-TM4SF4 axis promotes the progression of colorectal cancer by EMT and upregulating stem cell marker. Am J Cancer Res. 2022;12(3):1009-26. PMID: 35411242.

107.	Huang MY, Wang HM, Chang HJ, Hsiao CP, Wang JY, Lin SR. Overexpression of S100B, TM4SF4, and OLFM4 genes is correlated with liver metastasis in Taiwanese colorectal cancer patients. DNA Cell Biol. 2012;31(1):43-9. doi: 10.1089/dna.2011.1264. PMID: 22011044.

108.	Choi SI, Kim SY, Lee J, Cho EW, Kim IG. TM4SF4 overexpression in radiation-resistant lung carcinoma cells activates IGF1R via elevation of IGF1. Oncotarget. 2014;5(20):9823-37. doi: 10.18632/oncotarget.2450. PMID: 25344917.

109.	Choi SI, Kim SY, Lee JH, Kim JY, Cho EW, Kim IG. Osteopontin production by TM4SF4 signaling drives a positive feedback autocrine loop with the STAT3 pathway to maintain cancer stem cell-like properties in lung cancer cells. Oncotarget. 2017;8(60):101284-97. doi: 10.18632/oncotarget.21021. PMID: 29254164.

110.	Gamerith G, Rainer J, Huber JM, Hackl H, Trajanoski Z, Koeck S, et al. 3D-cultivation of NSCLC cell lines induce gene expression alterations of key cancer-associated pathways and mimic in-vivo conditions. Oncotarget. 2017;8(68):112647-61. doi: 10.18632/oncotarget.22636. PMID: 29348853.

111.	Jung K, Choi JS, Koo BM, Kim YJ, Song JY, Sung M, et al. TM4SF4 and LRRK2 Are Potential Therapeutic Targets in Lung and Breast Cancers through Outlier Analysis. Cancer Res Treat. 2021;53(1):9-24. doi: 10.4143/crt.2020.434. PMID: 32972043.

112.	Liang X, Peng J, Chen D, Tang L, Liu A, Fu Z, et al. Identification of novel hub genes and lncRNAs related to the prognosis and progression of pancreatic cancer by microarray and integrated bioinformatics analysis. Gland Surg. 2021;10(3):1104-17. doi: 10.21037/gs-21-151. PMID: 33842254.

113.	Liu J, Zheng M, Qi Y, Wang H, Liu M, Liu Q, et al. Lewis(y) antigen-mediated positive feedback loop induces and promotes chemotherapeutic resistance in ovarian cancer. Int J Oncol. 2018;53(4):1774-86. doi: 10.3892/ijo.2018.4496. PMID: 30066907.

120.	Westermann B. The mitochondria-plasma membrane contact site. Curr Opin Cell Biol. 2015;35:1-6. doi: 10.1016/j.ceb.2015.03.001. PMID: 25801776.

121.	Frieden M, Arnaudeau S, Castelbou C, Demaurex N. Subplasmalemmal mitochondria modulate the activity of plasma membrane Ca2+-ATPases. J Biol Chem. 2005;280(52):43198-208. doi: 10.1074/jbc.M510279200. PMID: 16216868.

122.	Fowler SL, Akins M, Zhou H, Figeys D, Bennett SA. The liver connexin32 interactome is a novel plasma membrane-mitochondrial signaling nexus. J Proteome Res. 2013;12(6):2597-610. doi: 10.1021/pr301166p. PMID: 23590695.

123.	Poolsri WA, Phokrai P, Suwankulanan S, Phakdeeto N, Phunsomboon P, Pekthong D, et al. Combination of Mitochondrial and Plasma Membrane Citrate Transporter Inhibitors Inhibits De Novo Lipogenesis Pathway and Triggers Apoptosis in Hepatocellular Carcinoma Cells. Biomed Res Int. 2018;2018:3683026. doi: 10.1155/2018/3683026. PMID: 29546056.

124.	Qiu J, Liu Z, Da L, Li Y, Xuan H, Lin Q, et al. Overexpression of the gene for transmembrane 4 superfamily member 4 accelerates liver damage in rats treated with CCl4. J Hepatol. 2007;46(2):266-75. doi: 10.1016/j.jhep.2006.08.011. PMID: 17069928.
