# Supplementary material for: Multiomics in silico analysis identifies TM4SF4 as a cell surface target in hepatocellular carcinoma
Source: PLoS One. 2025 Feb 25;20(2):e0307048. doi: 10.1371/journal.pone.0307048 (PMC11856526; doi:10.1371/journal.pone.0307048)
Supplement: Attachment — Submitted filename: Reviewer comments and recommendations.docx [file pone.0307048.s008.docx]

The authors of this article have applied a multi-omics data analysis of TM4SF4 as a cell surface target in hepatocellular carcinoma. This title is worthy of investigation and it is evident that lots of effort was put into writing this informative manuscript. However, some comments are mentioned below that need more clarification, explanation, or modification.

1. Please include the type of study in the title.
2. The abstract of the article is better to be structured.
3. The third paragraph of the introduction section needs to be concise. The amount of details that were provided may distract the attention of the reader from the main purpose of the study.
4. The authors have discussed some methods of the study in the last paragraph of the introduction section. Let them just remain in their appropriate place, i.e. the method section. You should include two things at the end of the introduction section: 1. the objective(s) of the study and 2. the novelty of the study
5. Please mention the type of the study in the method section.
6. Have you utilized any control group or not?
7. Have you provided any ethics approval number from a recognized institution? If yes, please mention it.
8. It is recommended to transfer all the website links to the references section.
9. Please mention the inclusion and exclusion criteria for the cases in your study.
10. Please mention the variables that were investigated in the methods section.
11. Please improve the cohesion and coherence of the discussion section and make it concise. It does not needed to report the findings of every previous study in a separate sentence. For instance, it is better to combine the findings of several similar previous studies and present them in one informative sentence in order to avoid redundancy.

Wish you luck and prosperity in revising the manuscript.
